# CircMALAT1 promotes cancer stem‐like properties and chemoresistance via regulating Musashi‐2/c‐Myc axis in esophageal squamous cell carcinoma

**DOI:** 10.1002/mco2.612

**Published:** 2024-06-14

**Authors:** Zitong Zhao, Yingni Deng, Jing Han, Liying Ma, Yumeng Zhu, Hua Zhang, Zhixu He, Yongmei Song

**Affiliations:** ^1^ Key Laboratory of Cancer and Microbiome State Key Laboratory of Molecular Oncology National Cancer Center/National Clinical Research Center for Cancer/Cancer Hospital Chinese Academy of Medical Sciences and Peking Union Medical College Beijing China; ^2^ Collaborative Innovation Center of Tissue Damage Repair and Regeneration Medicine Zunyi Medical University Zunyi China; ^3^ Department of Oncology The Fourth Hospital of Hebei Medical University Shijiazhuang Hebei China; ^4^ Beijing No.4 High School International Campus Beijing China; ^5^ School of Continuing Education Chinese Academy of Medical Sciences & Peking Union Medical College Beijing China

**Keywords:** cancer stem cell (CSC), circMALAT1, drug resistance, esophageal squamous cell carcinoma (ESCC), Musashi RNA binding protein 2 (MSI2)

## Abstract

The primary challenge in treating esophageal squamous cell carcinoma (ESCC) is resistance to chemotherapy. Cancer stem cell (CSC) is the root cause of tumor drug resistance. Therefore, targeting CSCs has been considered promising therapeutic strategy for tumor treatment. Here, we report that circMALAT1 was significantly upregulated in ESCC CSC‐like cells and primary tumors from ESCC patients. Clinically, there was a positive correlation between circMALAT1 expression and ESCC stage and lymph node metastasis, as well as poor prognosis for ESCC patients. In vitro and in vivo functional studies revealed that circMALAT1 promoted CSC‐like cells expansion, tumor growth, lung metastasis and drug resistance of ESCC. Mechanistically, circMALAT1 directly interacted with CSC‐functional protein Musashi RNA Binding Protein 2 (MSI2). CircMALAT1 inhibited MSI2 ubiquitination by preventing it from interacting with β‐transducin repeat containing protein (BTRC) E3 ubiquitin ligase. Also, circMALAT1 knockdown inhibited the expression of MSI2‐regulating CSC‐markers c‐Myc in ESCC. Collectively, circMALAT1 modulated the ubiquitination and degradation of the MSI2 protein signaling with ESCC CSCs and accelerated malignant progression of ESCC. CircMALAT1 has the potential to serve as a biomarker for drug resistance and as a target for therapy in CSCs within ESCC.

## Graphical abstract

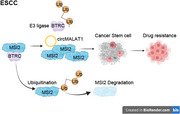

## 1 INTRODUCTION

According to GLOBOCAN 2020 data, there are 604,000 reported instances of esophageal cancer and 544,000 fatalities worldwide in 2020, placing it seventh and sixth in terms of all types of tumors.^1^ Approximately 85% (512,500 cases) of the new instances were diagnosed as esophageal squamous cell carcinoma (ESCC), while 15% (85,700 cases) were identified as esophageal adenocarcinoma.^1^, ^2^ The majority of ESCC cases are diagnosed in the middle to advanced stages, with chemotherapy being the primary treatment for advanced ESCC.^3^, ^4^ However, the effect of chemotherapy for ESCC is not ideal.^5^ Overall, ESCC receives poor treatment, with a 5‐year survival rate of only 10–30%.^1^, ^6^

Drug resistance is the main obstacle to treating tumors with chemotherapeutic drugs. Most clinical drug resistance is caused by activating development‐related signaling pathways, enhancing DNA repair abilities, as well as drug efflux mediated by ATP‐binding cassette transporters.^7^, ^8^, ^9^, ^10^, ^11^, ^12^ However, cancer stem cell (CSC) is the root cause of tumor drug resistance. Although it has become a consensus that CSCs exist in many kinds of tumors, why CSCs produce drug resistance, what is the mechanism of drug resistance, and the true characteristics of CSCs have not been well elucidated for a long time. For the treatment of tumor recurrence, identification of CSCs in various tumors as well as the microenvironment and biological processes necessary to maintain CSC characteristics are vital.

CircRNA is involved in a variety of normal and abnormal biological processes.^13^ Additionally, it is essential in the formation of tumors.^14^, ^15^ In contrast to linear RNA, circRNA has a closed ring structure. CircRNA, with its circular shape, exhibits increased stability in tissue and blood, making it resistant to degradation and offering numerous benefits as a molecular marker. A feature of circRNA that differentiates it from linear RNA and protein is its stability and enhanced protein expression, which makes it a great candidate for use in the development of vaccines, cancer immunotherapy, protein replacement therapy, and gene editing. In this regard, circRNA represents a promising RNA form in the development of RNA‐based drugs, which is expected to overturn personalized medical treatment.^16^, ^17^, ^18^ CircRNA has a diversity of biological functions, including protein and gene sponges, cell activity regulators, and protein translation templates.^19^, ^20^ A recent research study validated that circIPO11 enhances the self‐renewal ability of liver CSCs through the regulation of Hedgehog signaling pathways.^21^ However, the understanding of circRNA in CSC and drug resistance in ESCC remains largely a mystery.

This research involved conducting high‐throughput sequencing to confirm the high expression of circMALAT1 in CSC‐like cells and ESCC samples from patients. Previously study reported that circMALAT1 promoted self‐renewal of hepatocellular CSCs.^22^ CircMALAT1 is derived from chromosome11:65271199‐65272066, with a length of 867 nt, and the main gene is lncRNA MALAT1. CircMALAT1 inhibits the translation of paired box 5 (PAX5) by interacting with the coding sequence of PAX5 and ribosomes, while also increasing the expression of Janus kinase 2 (JAK2) by functioning as a sponge for miR‐6887‐3p.^22^ Our results demonstrate that circMALAT1 acts as a critical positive regulator in the control of tumor stemness and drug resistance of ESCC by binding to the CSC‐functional protein Musashi RNA Binding Protein 2 (MSI2). A new discovery was made regarding the circMALAT1 mechanism, which showed that circMALAT1 increased MSI2 expression by blocking the interaction between β‐transducin repeat containing protein (BTRC) E3 ubiquitin ligase and MSI2. In summary, circMALAT1 may be used for diagnostic biomarkers and therapeutic strategies to treat patients with ESCC.

## 2 RESULTS

### 2.1 CircMALAT1 expression was preferentially upregulated in ESCC CSC‐like cells

**FIGURE 1:**
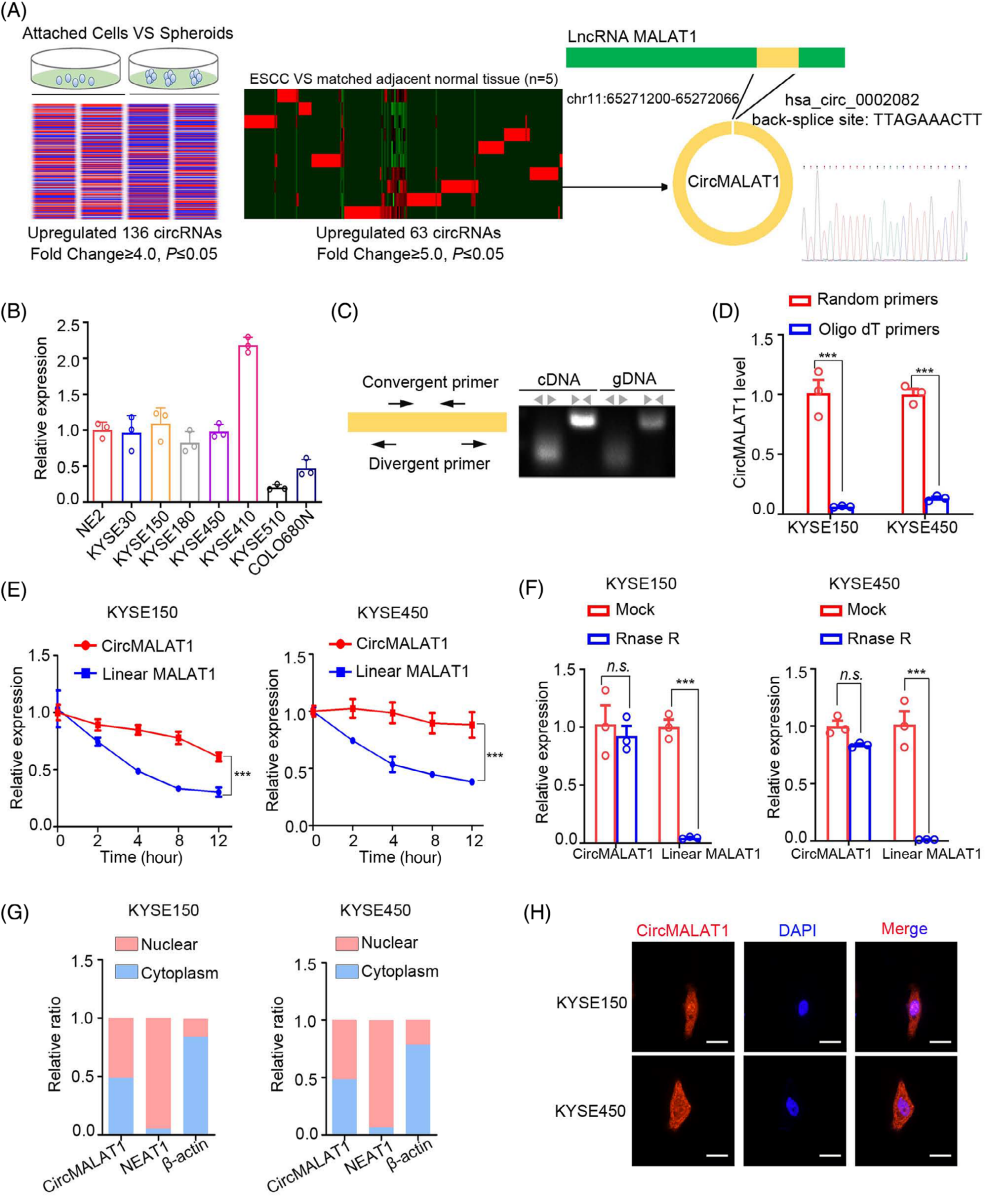
Circular RNA identification using RNA‐seq and characteristics of circMALAT1 in ESCC. (A) Schematic illustration of the experimental approach used to enrich esophageal squamous cell carcinoma (ESCC) cancer stem cell (CSC)‐like cells (left). Transcriptome profiling was performed on the chemo‐resistant spheroids, as well as their counterparts (attached cells cultured without cisplatin). Transcriptome sequencing was performed with five pairs of ESCC tissues compared with adjacent normal tissues. An overlapping of differentially expressed genes between the two datasets ruled out circMALAT1. Diagram showing the locus of circMALAT1 in the genome (middle). Schematic illustration showing that exon of human lncRNA MALAT1 circularize to form circMALAT1. Sanger sequencing has confirmed the back‐splicing site in circMALAT1 (right). (B) The expression of circMALAT1 in immortalized esophageal epithelium cell lines (NE2) and ESCC cells performed by quantitative real‐time PCR (qPCR). (C) PCR was performed to detect the existence of circMALAT1 and MALAT1 from cDNA and gDNA in KYSE150 using the divergent and convergent primers, respectively. Divergent primers amplified circMALAT1 in cDNA but not genomic DNA (gDNA). (D) qPCR analysis of circMALAT1 with different reverse transcription primers (random primers or oligo dT primers). (E) qPCR detected the expression of circMALAT1 and its linear transcript MALAT1 with actinomycin D (100 ng/mL) treatment. (F) qPCR detected the expression of circMALAT1 and MALAT1 with or without RNase R (2 U/µg RNA) treatment. (G) The subcellular fraction of circMALAT1 in cytoplasm or nucleus in KYSE150 and KYSE450 using qPCR. β‐Actin was used for cytoplasmic internal reference and lncRNA‐NEAT1 for nuclear internal reference. (H) The subcellular localization of circMALAT1 in KYSE150 and KYSE450 cells performed with FISH. Scale bar, 30 µm. The data are presented as the mean ± SD, ***p < 0.001.

To identify circRNAs involved in chemo‐resistance characteristics of CSC, we enriched the ESCC CSC‐like cells by inducing KYSE450 spheroid formation under chemotherapeutic conditions (cisplatin) and performed high‐throughput sequencing (GSA accession numbers: HRA007183), using a similar approach to that previously reported.^23^, ^24^ Based on a cutoff value of fourfold (*p* value ≤ 0.05), 136 circRNAs were determined to be highly expressed in the ESCC CSCs (Table S1). Then, high‐throughput sequencing was performed on five paired tissues of ESCC and adjacent tissues. Among the differentially expressed circRNAs, 63 circRNAs were upregulated in ESCC tissues than adjacent tissues (fold change ≥ 5.0 and *p* value ≤ 0.05; Table S2). Comprehensive analysis of the above two sets of data, only circMALAT1 was upregulated expressed not only in ESCC tissues, but also in ESCC CSC‐like cells, so we chose circMALAT1 for further study (Figure 1A). Additionally, circMALAT1 was overexpressed in ESCC cell lines when compared with immortalized esophageal epithelial cells NE2 (Figure 1B). Convergent and divergent primers were designed to determine the ring structure of circMALAT1 (Figure 1C,D). The actinomycin D RNA stability and RNase R digestion assays confirmed the stability of circMALAT1 compared with MALAT1 (Figure 1E,F). The quantitative real‐time PCR (qPCR) and Fluorescence in situ hybridization results showed that the distribution of circMALAT1 was both in nucleus and cytoplasm (Figure 1G,H).

### 2.2 CircMALAT1 enhances CSC‐like properties of ESCC cells

**FIGURE 2:**
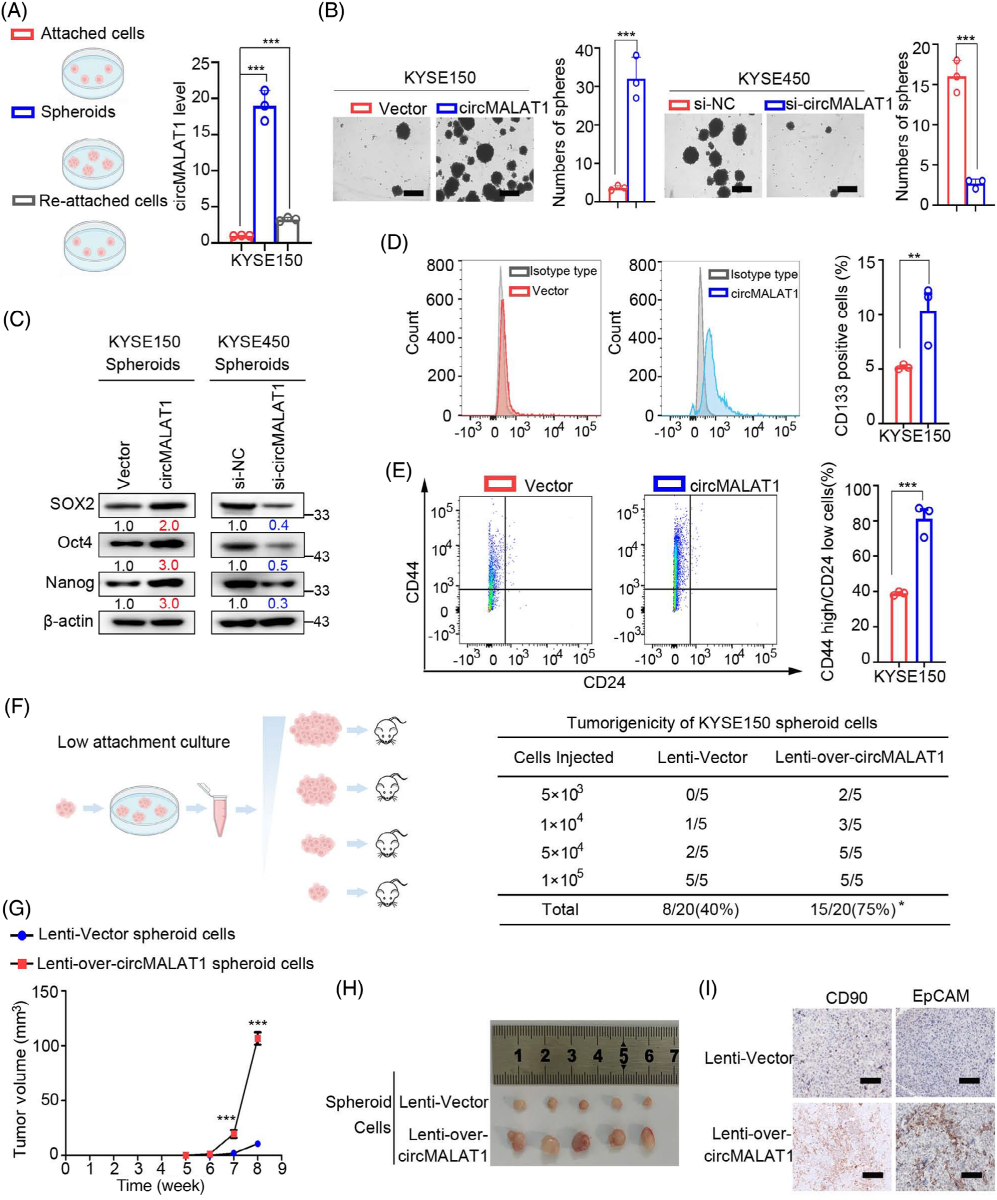
Upregulation of circMALAT1 enhances CSC‐like features of ESCC cells in vitro and in vivo. (A) Expression of circMALAT1 in ESCC attached cells, spheroids, and spheroids‐reattached cells was examined by qPCR. (B) Representative images of ESCC spheroids generated from KYSE150/KYSE450 overexpressing or knockdown of circMALAT1 and corresponding control cells. Scale bar, 500 µm. (C) Western blot analysis of the stemness‐associated transcription factors (SOX2, OCT4, Nanog) in spheroids generated from KYSE150/KYSE450 overexpressing or knockdown of circMALAT1 and corresponding control cells. (D and E) Flow cytometric analysis of the CD133 and CD44^high^/CD24^low^ expression level in spheroids generated from KYSE150 overexpressing circMALAT1 and corresponding control cells. (F–I) Animal models for the in vivo limiting dilution assay to assess the role of circMALAT1 in ESCC tumor initiation. (F) Schematic illustration of the experimental approach applied to assess the tumorigenicity and ESCC CSCs frequency in vivo. KYSE150 infected with lentivirus‐mediated Lenti‐vector and Lenti‐over‐circMALAT1 (left). NOD‐SCID mice were subcutaneously inoculated with ESCC cells dissociated from either KYSE150 vector spheroids or circMALAT1 spheroids and tumorigenicity was assessed two months after inoculation (right). (G) Tumor growth curve analysis of mice bearing Lenti‐over‐circMALAT1 spheroid cells (1 × 10^5^ cells) compared with mice bearing control cells. (H) The images of the ultimately formed tumors (1 × 10^5^ cells). (I) Representative images of immunohistochemical staining of CD90 and EpCAM in xenografted tumors. Scale bar, 100 µm. The data are presented as the mean ± SD, **p < 0.01, ***p < 0.001.

To uncover the role of circMALAT1 for CSC‐like properties in ESCC, the RNAs of KYSE150 and KYSE450 in attached, spheroids, and re‐attached states were collected. qPCR was used to detect circMALAT1 expression. The results suggested that spheroids exhibited significantly higher levels of circMALAT1 expression than attached cells. Intriguingly, parallel to differentiation, circMALAT1 may be partially restored during reattachment (Figures 2A and S1A). Spheroid formation was enhanced in circMALAT1 overexpressing KYSE150/KYSE450 cells and attenuated in circMALAT1 knockdown KYSE150/KYSE450 cells (Figures 2B and S1B,1C). ESCC spheroids from circMALAT1 overexpressed cells showed increased expression of stemness‐associated transcription factors (SOX2, Oct4, and Nanog) compared with control cells, while ESCC spheroids with circMALAT1 knockdown exhibited decreased levels of these transcription factors (Figures 2C and S1D). Moreover, overexpressed circMALAT1 spheroids contained significantly higher CD44^high^/CD24^low^ and CD133 positive cells in comparison with vectors (Figures 2D,E and S1E).

To study the impact of circMALAT1 on ESCC CSC expansion, dissociated ESCC cells from cultured spheroids were subjected to a limiting dilution assay (LDA). Compared with control spheroids, circMALAT1 overexpressing spheroids exhibited higher tumorigenicity and ESCC frequency (Figure 2F). Further study revealed that ESCC cells from circMALAT1 overexpressing spheroids showed enhanced xenografted tumor growth (Figure 2G), as well as increased tumor size in vivo (Figure 2H). The above results suggest the promoting role of circMALAT1 in ESCC propagation and ESCC progression. Meanwhile, circMALAT1 overexpressing spheroid‐formed xenografts exhibited increased EpCAM+ and CD90+ ESCC CSCs compared with control xenografts (Figures 2I and S1F), highlighting that circMALAT1 enhanced the CSC‐like properties of ESCC cells.

### 2.3 CircMALAT1 is upregulated in ESCC and its higher level predicts lymph node metastasis and poor prognosis

**FIGURE 3:**
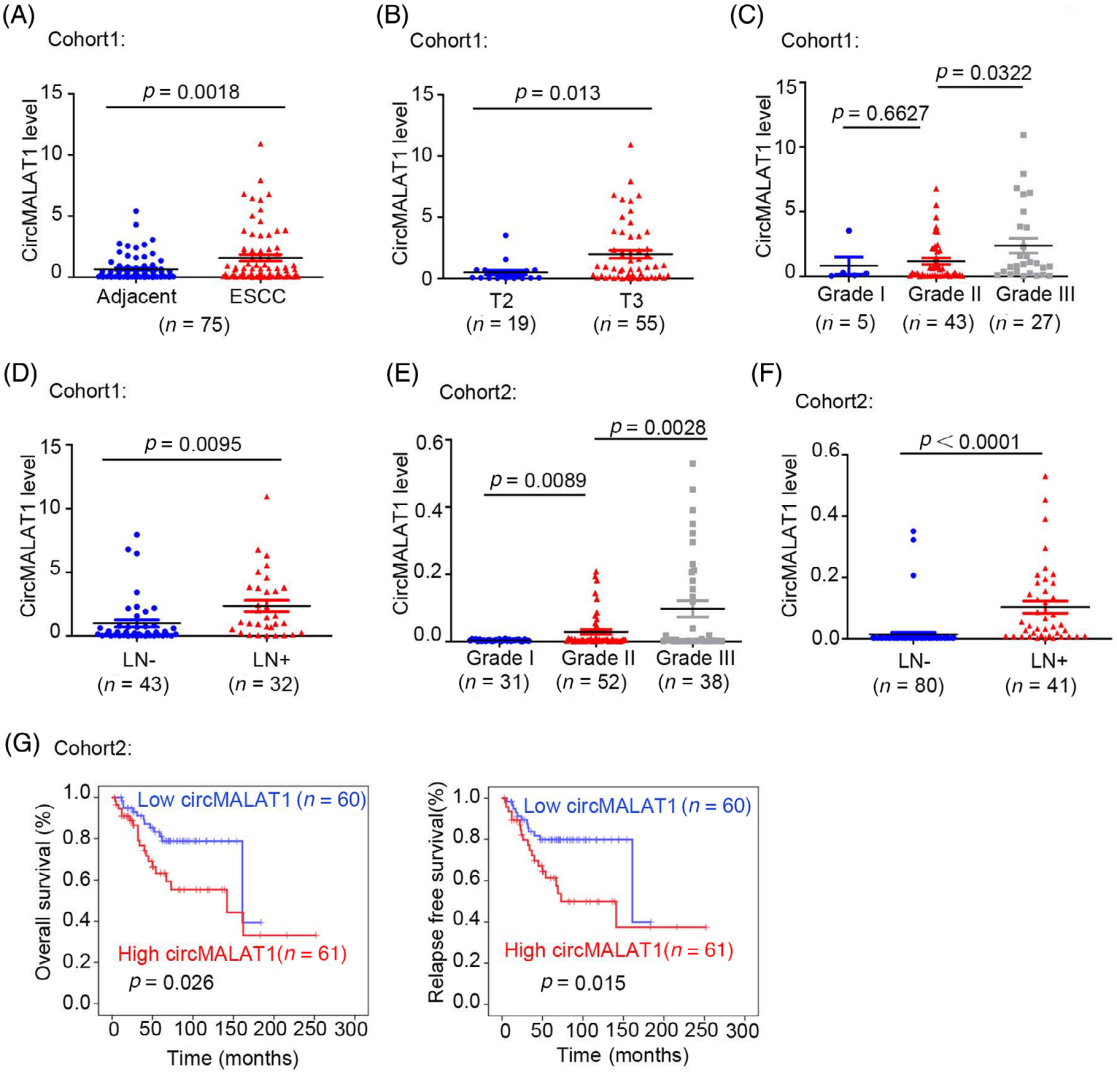
CircMALAT1 is overexpressed in ESCC and is associated with lymphatic metastasis and poor survival in advanced ESCC. (A–D) qPCR of the circMALAT1 levels in 75 pairs of ESCC tissues and their corresponding noncancerous samples (cohort1). The results were normalized to the endogenous β‐actin RNA control. (A) The level of circMALAT1 was significantly higher in ESCC samples than in their corresponding noncancerous samples in cohort1. (B) The level of circMALAT1 was significantly higher in T3 stage ESCC samples than in T2 stage ESCC samples in cohort1. (C) The level of circMALAT1 was significantly higher in patients with grade III ESCC than in the other tumor samples in cohort1. (D) The level of circMALAT1 was significantly higher in ESCC patients with lymphatic metastasis than in ESCC patients without lymphatic metastasis in cohort1. (E–G) qPCR of the circMALAT1 levels in 121 ESCC samples (cohort2). The results were normalized to the endogenous β‐actin RNA control. (E) The level of circMALAT1 was significantly higher in patients with grade III ESCC than in the other tumor samples in cohort2. (F) The level of circMALAT1 was significantly higher in ESCC patients with lymphatic metastasis than in ESCC patients without lymphatic metastasis in cohort2. (G) Kaplan–Meier curves for ESCC patients with low versus high circMALAT1 expression levels in cohort2; log‐rank test.

CircMALAT1 expression was determined in 75 paired ESCC tumor and adjacent nontissue samples from patients (cohort1). We observed that circMALAT1 expression is significantly elevated in tumor tissues compared with nontumor tissues adjacent to tumors (Figure 3A). Further, the analysis of the pathological parameters and circMALAT1 expression level was carried out. The findings indicated a positive correlation between circMALAT1 expression levels and T stage, differentiation stages, and lymph node metastasis, as shown in Figure 3B–D  and Table S3. In cohort2, it was observed that the high levels of circMALAT1 expression were correlated with differentiation stages and lymph node metastasis, as indicated in Figure 3E,F and Table S4. A strong correlation was found between increased levels of circMALTA1 and decreased overall survival time and relapse survival time in patients with ESCC (Figure 3G).

### 2.4 CircMALAT1 promotes the aggressive behavior of ESCC cells in vitro

**FIGURE 4:**
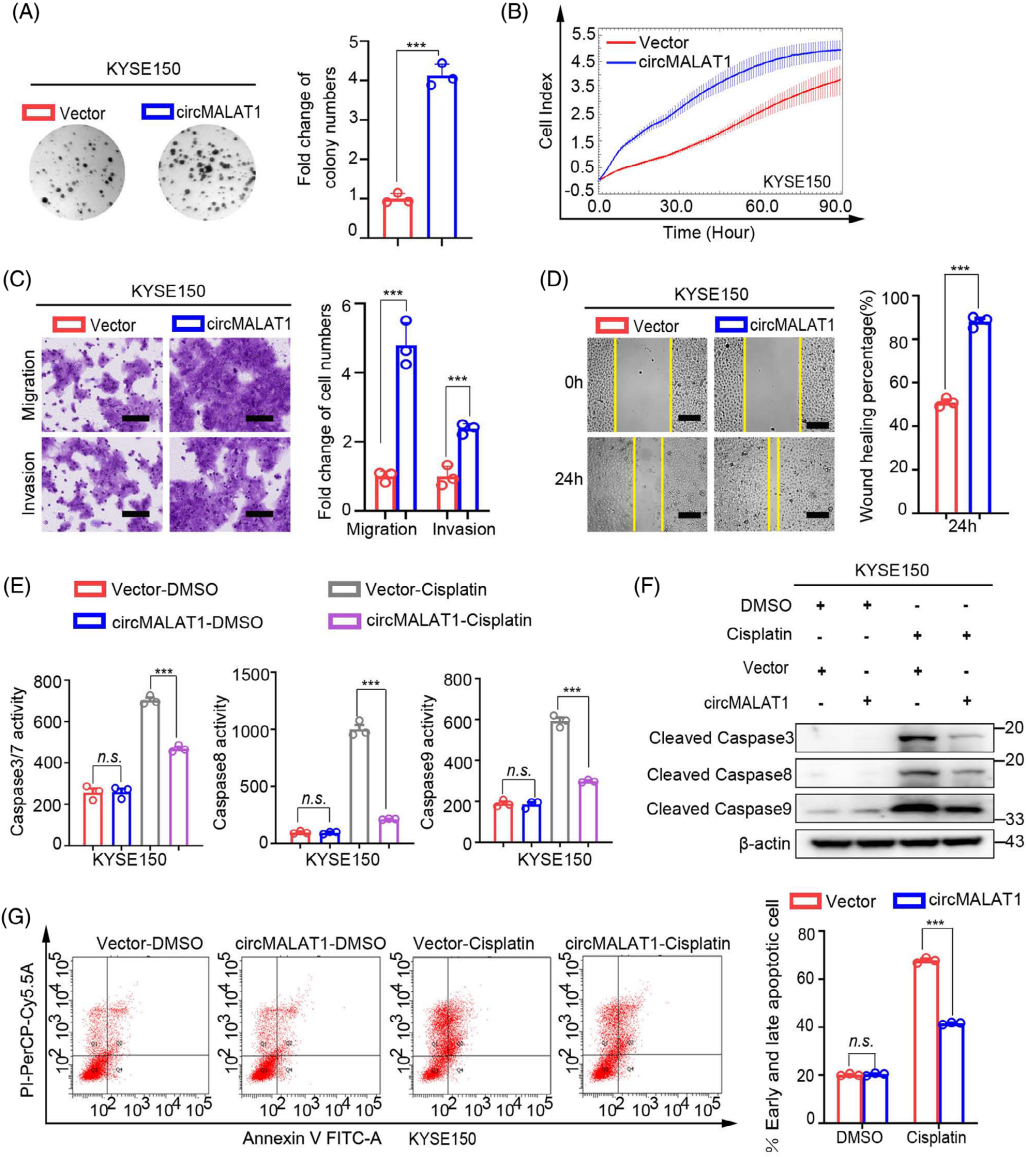
CircMALAT1 promotes cell proliferation, migration, invasion and impedes apoptosis in ESCC cells. The KYSE150 cells were transfected with circMALAT1 plasmid and corresponding controls. (A) Cell proliferation ability was evaluated by colony formation. (B) Growth ability analysis with the xCELLigence Real‐Time Cell Analyzer (RTCA)‐MP system. (C) Transwell assays were used to assess the migration and invasion abilities of ESCC cells. Scale bar, 100 µm. (D) Wound‐healing assays were performed to measure the migration abilities of ESCC cells. Scale bar, 200 µm. (E) Caspase3/7, caspase8, and caspase9 activity was assessed using the fluorogenic substrate after the indicated cells were treated with cisplatin (20 µg/mL) for 24 h. (F) Western blot analysis of apoptosis‐related protein levels after the indicated cells were treated with cisplatin (20 µg/mL) for 24 h. (G) Flow cytometry analysis of apoptosis (Annexin V/PI) cells after the indicated cells were treated with cisplatin (20 µg/mL) for 24 h. The data are presented as the mean ± SD, ***p < 0.001.

**FIGURE 5:**
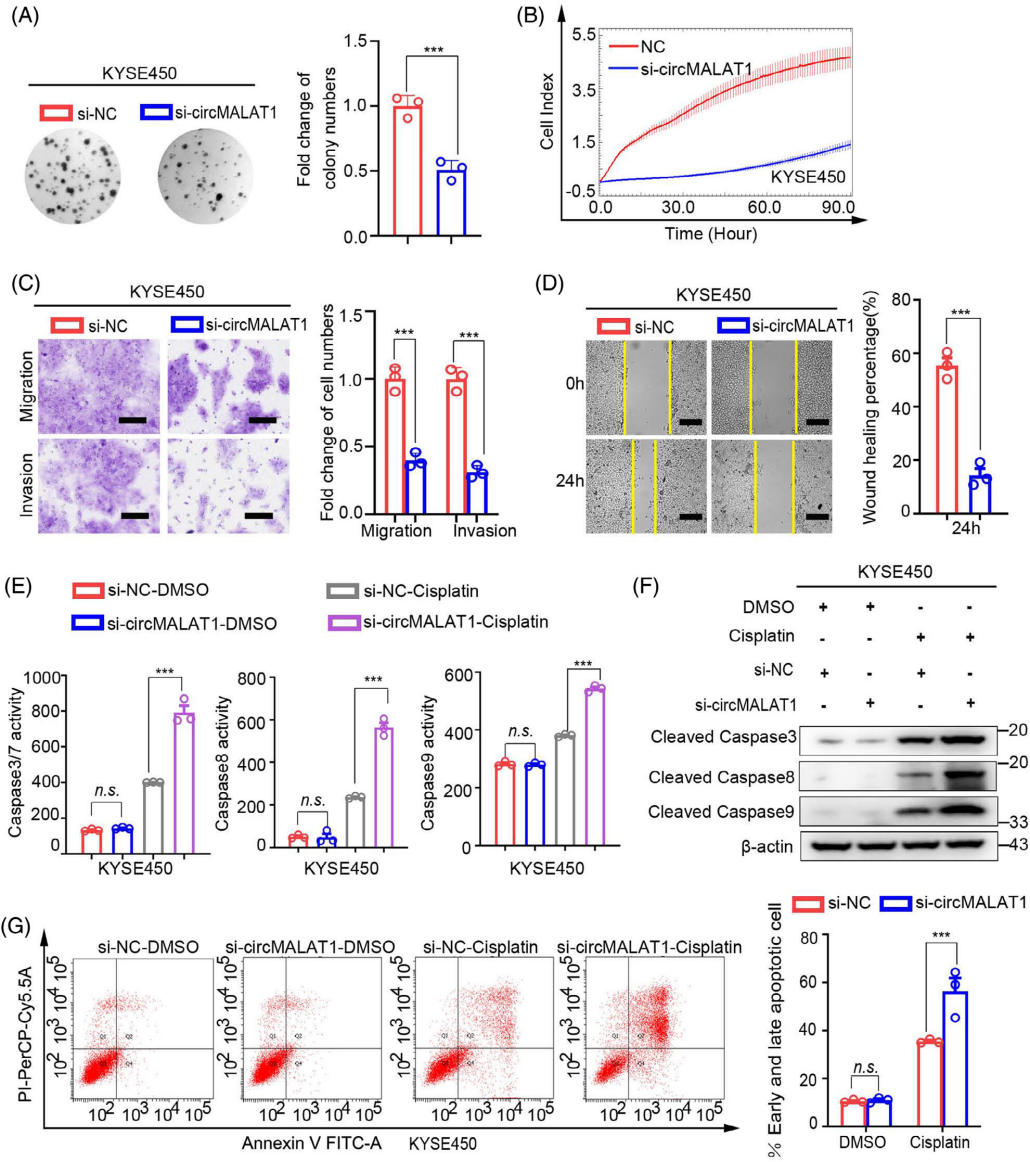
Silencing of circMALAT1 inhibits the proliferation, migration, invasion and induces apoptosis in ESCC cells. The KYSE450 cells were transfected with circMALAT1‐specific siRNA and corresponding controls. (A) Cell proliferation ability was evaluated by colony formation. (B) Growth ability analysis with the xCELLigence Real‐Time Cell Analyzer (RTCA)‐MP system. (C) Transwell assays were used to measure the migration and invasion abilities of ESCC cells. Scale bar, 100 µm. (D) Wound‐healing assays were performed to assess the migration abilities of ESCC cells. Scale bar, 200 µm. (E) Caspase3/7, caspase8, and caspase9 activity was assessed using the fluorogenic substrate after the indicated cells were treated with cisplatin (20 µg/mL) for 24 h. (F) Western blot analysis of apoptosis‐related protein levels after the indicated cells were treated with cisplatin (20 µg/mL) for 24 h. (G) Flow cytometry analysis of apoptosis (Annexin V/PI) cells after the indicated cells were treated with cisplatin (20 µg/mL) for 24 h. The data are presented as the mean ± SD, ***p < 0.001.

To further explore the biological roles of circMALAT1, we performed gain‐of‐ and loss‐of‐function studies. As shown in Figures 4A–D and S2A–D, the results of phenotype experiments of KYSE150 and KYSE450 cells showed that the colony formation ability, proliferation, migration, and invasion of ESCC cells were significantly enhanced following overexpression of exogenous circMALAT1. Additionally, overexpression circMALAT1 enhanced the drug resistance of cells to cisplatin (Figures 4E–G and S2E–G). Moreover, knocking down circMALAT1 weakened the colony formation ability, proliferation, migration, invasion, and increased the sensitivity of KYSE150 and KYSE450 cells to cisplatin (Figures 5A–G and S3A–G). This indicate that circMALAT1 plays a cancer‐promoting role in ESCCs.

### 2.5 CircMALAT1 promotes ESCC growth and lung metastasis in vivo

**FIGURE 6:**
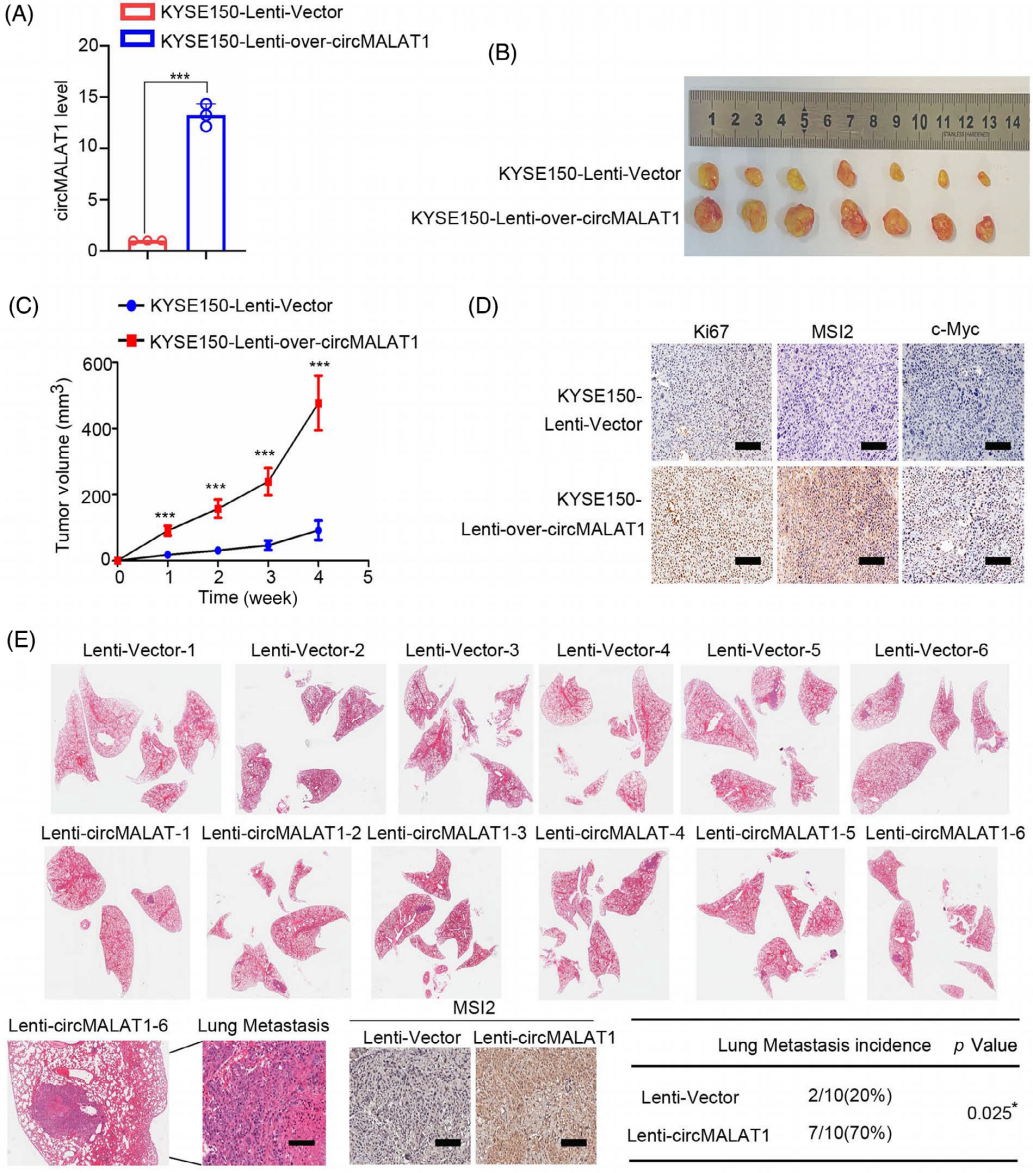
CircMALAT1 promotes the growth, lung metastasis of ESCC in vivo. (A) KYSE150 cells were stably infected with lentiviral vectors expressing control (Lenti‐Vector) or circMALAT1 (Lenti‐over‐circMALAT1), and the circMALAT1 levels were analyzed by qPCR. (B–D) The top backs of the mice were injected subcutaneously with 5 × 10^5^ KYSE150 Lenti‐Vector or Lenti‐circMALAT1 cells in 100 µL. Tumors induced by Lenti‐circMALAT1 cells and control tumors were measured weekly in nude mice (*n* = 7). (B) The images of the ultimately formed tumors. (C) Tumor growth curve analysis of mice bearing Lenti‐circMALAT1 cells compared with mice bearing control cells. (D) Representative Immunohistochemistry (IHC) staining of Ki67, MSI2, c‐Myc in KYSE150 xenografts with the indicated treatments. Scale bar, 100 µm. (E) Rate of lung metastasis after the tail vein injection of Lenti‐Vector and Lenti‐over‐circMALAT1 cells. Lung metastasis of representative tumors was determined by HE analysis. Representative IHC staining of MSI2 in lung metastasis. Scale bar, 100 µm. The data are presented as the mean ± SD, *p < 0.05, ***p < 0.001.

To investigate the biological functions of circMALAT1 in ESCC in vivo, stably overexpressed circMALAT1 in KYSE150 cell was established via lentivirus system (Figure 6A). A nude mouse subcutaneous xenograft model was applied. KYSE150 cells with circMALAT1 overexpression had a significantly higher tumor growth rate and tumor volume (Figure 6B,C). Histologic examination of Ki67 showed that tumors generated from circMALAT1‐overexpressing KYSE150 cells had increased cell proliferation indices (Figure 6D). In addition, tail vein injection mouse model was established. In comparison with the control group, circMALAT1 overexpression significantly facilitated lung metastases in KYSE150 cells (Figure 6E). Immunohistochemistry (IHC) result indicated that the lung metastases with circMALAT1 overexpression had a significantly higher MSI2 level (Figure 6E). Collectively, these data reveal that circMALAT1 overexpression promoted ESCC growth and metastatic potential in vivo.

### 2.6 CircMALAT1 stabilizes MSI2 by blocking BTRC E3 Ub ligase‐mediated ubiquitination

**FIGURE 7:**
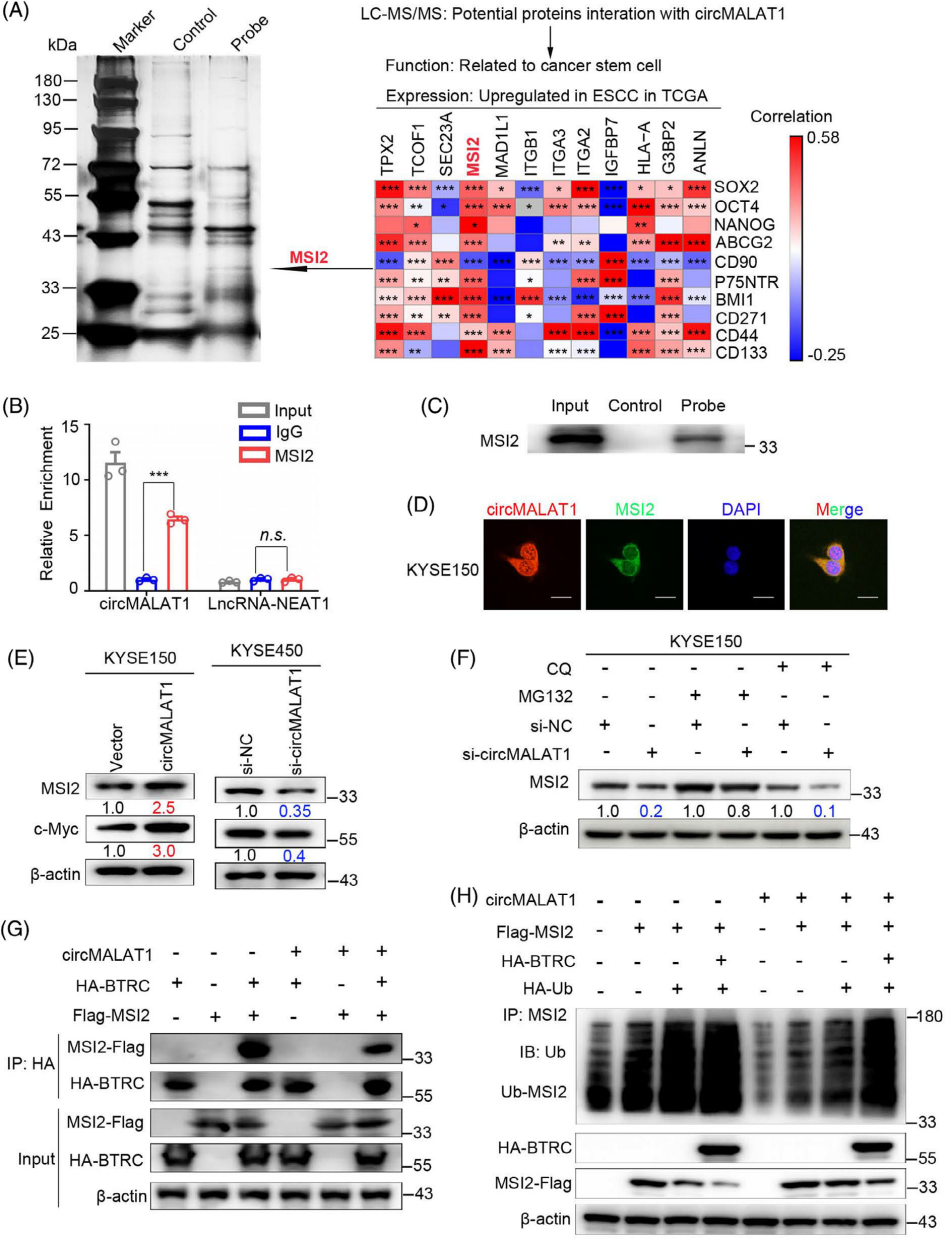
CircMALAT1 enhances MSI2 stability by disrupting the interactions between MSI2 and BTRC E3 ubiquitin ligase. (A) LC–MS/MS were performed to identify circMALAT1 interacting proteins in KYSE150 after pull‐down with biotinylated circMALAT1 probe and control (left). Correlation between reported ESCC CSC markers (SOX2, OCT4, NANOG, ABCG2, CD90, BMI1, CD271, CD44, and CD133) and mRNA level of candidate protein interaction with circMALAT1(Fold change of probe/control ≥ 5.0) in ESCC in TCGA database. (B) RNA immunoprecipitation (RIP) assay in KYSE150 confirmed that circMALAT1 could be enriched by MSI2. LncRNA‐NEAT1 was used as the negative control. (C) The interaction between circMALAT1 and MSI2 was confirmed by RNA pull‐down assays and Western blot analysis. (D) Colocalization analysis of circMALAT1 and MSI2 using protein IF and RNA FISH assays, respectively. Scale bar, 30 µm. (E) Western blot detection of MSI2 protein half‐life in KYSE150 cells transfected with the circMALAT1 and indicated control and treated with cycloheximide (CHX) (100 µg/mL) for the indicated timepoints. (F) Western blot of MSI2 levels in the circMALAT1‐siRNA cells and corresponding control cells were treated with proteasome inhibitor MG132 (10 µM) or chloroquine (10 µM) for 12 h. (G) IP was performed with anti‐HA antibody in KYSE150 cells transfected with HA‐BTRC and Flag‐MSI2 plasmids in the presence or absence of circMALAT1, followed by immunoblotting (IB) with the indicated antibodies. The cells were treated with MG132 (10 µM) for 12 h before harvesting. (H) KYSE150 cells were cotransfected with the indicated plasmids and treated with MG132 (10 µM) for 12 h before collection. MSI2 pull‐down experiments were conducted, and the samples were analyzed using western blotting with the indicated antibodies. The data are presented as the mean ± SD, *p < 0.05, **p < 0.01, ***p < 0.001.

To further elucidate the underlying mechanism of circMALAT1 in ESCC, RNA pulldown assay coupled with mass spectrometry were performed to identify circMALAT1‐associated proteins. There were 306 proteins that were specifically pulled down by circMALAT1 probe (Table S5). We screened 12 out of the 306 proteins according to two criteria: (1) The mRNA of the protein is abnormally high expressed in ESCC in TCGA database; (2) the function of the protein is related to CSC. Subsequently, we analyzed the correlation between the reported ESCC CSC markers (SOX2, OCT4, Nanog, ABCG2, CD90, BMI1, P75NTR, CD271, CD44, CD133) and the mRNA level of these 12 proteins in ESCC in TCGA database, and found that MSI2 had the highest correlation with the ESCC CSC markers (Figure 7A). The MSI2 belongs to the Musashi family, which expressed in neural and hematopoietic progenitor cells. This RNA‐binding protein (RBP) has the ability to control the growth and development of progenitor cells.^25^ It is also capable of imparting and maintaining stemness.^26^ MSI2 successfully precipitated circMALAT1 in oncosphere lysates using RNA immunoprecipitation (RIP) assay, as shown in Figure 7B. The interaction of circMALAT1 with MSI2 was further validated by RNA pulldown assay coupled with western blot (Figure 7C). Colocalization of circMALAT1 with MSI2 was further confirmed by immunofluorescence staining in KYSE150 and KYSE450 cells (Figures 7D and S4A).

To further explore the effect of circMALAT1 on MSI2, we detected MSI2 protein expression in ESCC cells with silenced circMALAT1 or overexpressed circMALAT1. Results showed that knockdown of circMALAT1 dramatically downregulated MSI2 protein and its downstream protein c‐Myc level, while overexpression of circMALAT1 remarkably upregulated MSI2 and c‐Myc protein level (Figures 7E and S4B). However, the expression level of MSI2 mRNA was not affected by the overexpression of circMALAT1 in ESCC cells (Figure S4C–E). Therefore, circMALAT1 is mainly responsible for regulating MSI2 protein expression.

To determine whether circMALAT1 regulates MSI2 protein stability, we further performed cycloheximide (CHX) assay. The findings indicated that the MSI2 protein's half‐life was extended in the circMALAT1 overexpressing group compared with the control group, as demonstrated in Figure S4F. We next found that pretreatment with MG132 prevented circMALAT1 knockdown‐induced degradation of MSI2 protein in ESCC cells (Figure 7F). Moreover, there is no significant effect on circMALAT1 knockdown‐induced degradation of MSI2 protein in ESCC cells, which were pretreated with chloroquine (Figures 7F and S4G). Therefore, the regulation of MSI2 degradation by circMALAT1 is not through lysosomal pathway but ubiquitin pathway. We then cotransfected circMALAT1 plasmid or siRNA for circMALAT1 with ubiquitin into ESCC cells and treated the transfected cells with MG132. Co‐immunoprecipitation (Co‐IP) assay revealed that circMALAT1 overexpression remarkably decreased the levels of ubiquitinated MSI2, while knocking down circMALAT1 significantly increased the levels of ubiquitinated MSI2 (Figure S4H). Previously study reported that BTRC E3 mediated ubiquitination degradation of MSI2.^25^ Notably, due to its ability to interrupt the interaction between MSI2 and BTRC, circMALAT1 was involved in the degradation of MSI2 by BTRC (Figure 7G) and block the decrease of MSI2 level caused by BTRC overexpression (Figure S4I). More importantly, exogenous BTRC promoted MSI2 ubiquitination, whereas overexpression circMALAT1 prevented this ubiquitination (Figure 7H). Therefore, we speculate that circMALAT1 binds to MSI2 specifically, inhibiting its interaction with BTRC, thus preventing the degradation of MSI2 by E3 Ub‐mediated ubiquitination.

### 2.7 CircMALAT1 initiates MSI2/c‐myc signaling activation for ESCC stemness and malignant phenotype

**FIGURE 8:**
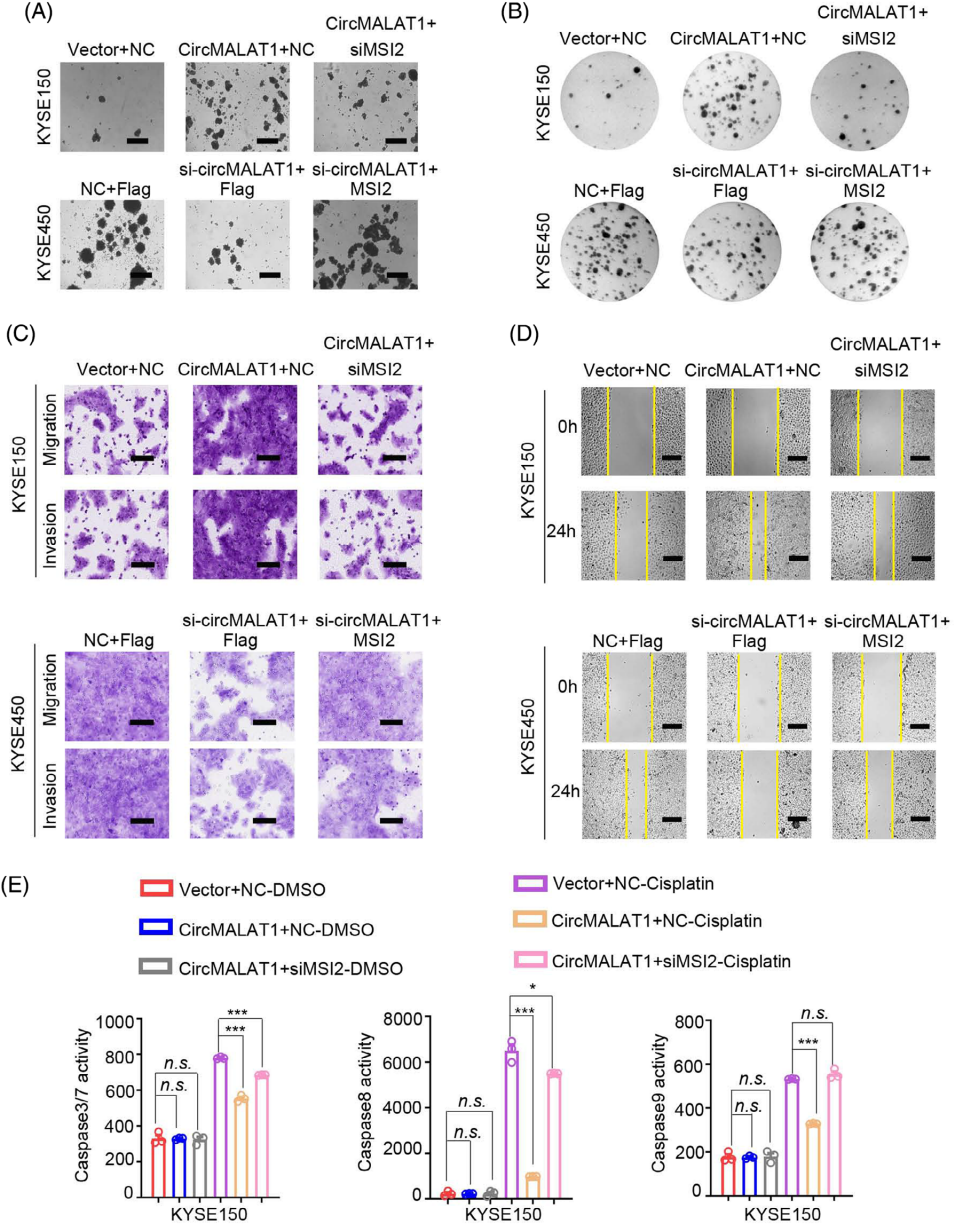
CircMALAT1 promotes CSC‐like properties and malignant progression by MSI2/c‐Myc signaling pathway in ESCC. The KYSE150 cells were transfected with circMALAT1 plasmid or cotransfected with si‐MSI2. The KYSE450 cells were transfected with si‐circMALAT1 or cotransfected with MSI2 plasmid. (A) Representative image of spheroids generated from KYSE150/KYSE450 cells. The number of spheroids was counted and compared. Scale bar, 500 µm. (B) Cell proliferation ability was evaluated by colony formation. (C) Transwell assays were used to measure the migration and invasion abilities of ESCC cells. Scale bar, 100 µm. (D) Wound‐healing assays were performed to assess the migration abilities of ESCC cells. Scale bar, 200 µm. (E) Caspase3/7, caspase8, and caspase9 activity was assessed using the fluorogenic substrate after the indicated cells were treated with cisplatin (20 µg/mL) for 24 h. The data are presented as the mean ± SD, *p < 0.05, **p < 0.01, ***p < 0.001.

The aforementioned results demonstrate that circMALAT1 interacts with MSI2 and positively regulates MSI2 protein level. To further investigate the physiological relevance of circMALAT1 and MSI2 in ESCC, we performed rescue experiments. We overexpressed circMALAT1 while knocking down MSI2 in KYSE150/KYSE450, and observed that the ectopic expression of MSI2 attenuated circMALAT1 overexpression‐induced mammosphere formation ability, colony formation ability, proliferation, migration and invasion, and drug resistance of cells to cisplatin (Figures 8A–E and S5–S10). Furthermore, we simultaneously expressed siRNA targeting circMALAT1 and MSI2 plasmid, revealing that increased MSI2 expression effectively counteracts the negative impact on mammosphere formation, colony formation, proliferation, migration, invasion, and cisplatin resistance in ESCC cells resulting from circMALAT1 knockdown (Figures 8A–E and S5–S10). Collectively, these data suggest that circMALAT1 exhibited an oncogenic effect by stabilizing MSI2 proteins, which augments its function leading to the CSCs and is responsible for cisplatin resistance of ESCC.

## 3 DISCUSSION

Tumor epigenetics elucidates a succession of heritable alterations in gene expression that are not induced by modifications in DNA sequence.^27^ Epigenetic mechanisms such as abnormal DNA methylation, histone modification, chromatin remodeling, mRNA transcription, and noncoding RNA expression play a crucial role in the development and advancement of tumors.^28^, ^29^, ^30^ This study demonstrated the importance of circMALAT1 in drug resistance of CSCs in ESCC both in vitro and in vivo. The research we conducted demonstrated that circMALAT1 hinders the proteasomal degradation of MSI2 by blocking the connection between MSI2 and BTRC E3 ubiquitin ligase. Additionally, circMALAT1 positively regulates MSI2/c‐Myc‐mediated stemness, chemoresistance of ESCC.

In spite of the fact that surgery, radiotherapy, and chemotherapy can reduce tumor size and prolong life, tumor recurrence and multidrug resistance remain major obstacles to the treatment of tumor. According to CSCs theory, tumor is a heterogeneous cell population, and there are a small number of CSC‐like cells in tumors. This group of CSC‐like cells are similar to embryonic stem cells and have unlimited self‐renewal and unlimited proliferation ability, which regulate the occurrence and development of tumor.^31^, ^32^, ^33^ Conventional radiotherapy and chemotherapy target ordinary tumor cells, but CSCs are not susceptible. At the same time, meanwhile, radiotherapy and chemotherapy stimulated CSCs and enriched them by altering the microenvironment. These lead to the further enhancement of the ability of tumor proliferation, invasion, and metastasis, and lead to stronger resistance to chemotherapeutic drugs, reduced sensitivity to drugs, or even no response, resulting in tumor recurrence.^34^, ^35^ In view of the fact that CSCs contributes significantly to drug resistance, new ideas for treating tumors can be developed by studying the mechanism of CSC resistance. Studies have shown that CSCs primarily contribute to resistance to cancer drugs by utilizing their unique biological features and the protective tumor microenvironment. This includes mechanisms like cell cycle arrest, DNA damage repair, drug efflux, and epithelial‐mesenchymal transition. Additionally, factors such as hypoxia, cancer‐associated fibroblasts, and chronic inflammation in the tumor microenvironment help maintain the stem cell properties of CSCs, making it harder to treat tumors effectively.^35^, ^36^ The research on the mechanism of drug resistance of CSCs can guide the development of new targeted therapy for CSCs. Combining targeted therapy for CSCs with conventional treatment methods can eradicate both CSCs and tumor cells simultaneously, enhancing the overall efficacy of tumor treatment.

Research on ESCC CSCs is limited in comparison with CSCs from other types of tumors, and there is a lack of identified cell surface markers specific to ESCC CSCs. Oct3, Oct4, SOX2, Nanog, and c‐Myc are all part of the group of stem cell transcriptional regulatory factors and serve as markers for CSCs; however, they are not exclusive markers for ESCC CSCs. Several research studies have been conducted on the control of ESCC CSCs development, with certain essential molecules overseeing ESCC stem cell characteristics through involvement in traditional stem cell signaling pathways such as Hippo, Notch, Hedgehog, and Wnt/β‐catenin signaling pathway.^37^, ^38^, ^39^, ^40^, ^41^, ^42^ Reports indicate that ribosomal S6 protein kinase 4 activates the β‐catenin signaling pathway by phosphorylating GSK‐3β directly, leading to the enhancement of CSC characteristics and resistance to radiation in ESCC.^42^ According to our data, circMALAT1 was preferentially upregulated in ESCC CSC‐like traits and higher circMALAT1 level facilitated ESCC initiation and progression. Furthermore, circMALAT1 is a positive regulator for MSI2.

Musashi family is a class of evolutionarily conserved RBPs. MSI2 belongs to Musashi family. A wide range of RNA post‐transcriptional modifications are carried out by RBPs, including RNA splicing, editing and alternative polyadenylation.^43^ MSI2 can regulate not only the translation of target mRNAs, but also the stability of target mRNAs.^44^ MSI2 is capable of preserving the undifferentiated state and self‐renewal capacity of various types of stem cells.^45^, ^46^ It used to be thought that MSI2 was widely expressed in stem cells and hematological tumors. Recent evidence has increasingly shown that MSI2 plays a role in the progression of solid tumors by promoting CSC‐like characteristics and boosting tumor growth, spread, metastasis, and resistance to drugs. MSI2 primarily operates through various signal pathways including TGF‐β/SMAD3, Akt/mTOR, JAK/STAT, Wnt/β‐catenin, Numb, and related pathways such as Notch, p53, and Hedgehog.^46^, ^47^, ^48^, ^49^, ^50^ MSI2 could inhibit the translation of Numb mRNA,^49^ it can also enhance c‐Myc mRNA stability.^25^ In our study, we found circMALAT1 directly interacted with MSI2, and protected MSI2 from degradation through preventing its interaction with BTRC E3 ubiquitin ligase.

However, there are still three important limitations to the study. We did not detect and compare the expression of circMALAT1 in chemotherapy‐sensitive and chemotherapy‐resistant patients with advanced ESCC. It will be better to use resistant cell lines and in vivo resistant model for the study on drug resistance of tumor, but we did not use the models. Besides interacted with MSI2, the other underlying mechanisms of how circMALAT1 regulating CSCs in ESCC were still ambiguous. These need to be further exploration.

Taken together, we presented a new paradigm in which circMALAT1 acts as a novel link between MSI2/c‐Myc signaling and ESCC CSCs. Our study expands the understanding of circRNA function in ESCC CSCs and ESCC pathogenesis and suggests that circMALAT1 is a biomarker that can predict prognosis and might even be used to treat ESCC CSCs.

## 4 MATERIALS AND METHODS

### 4.1 Patients and specimen collection

A total of 75 fresh ESCC specimen pairs and their corresponding adjacent paracancerous normal tissues, along with 121 FFPE tissue specimens from ESCC patients, were collected from the National Cancer Center/National Clinical Research Center for Cancer/Cancer Hospital, Chinese Academy of Medical Sciences and Peking Union Medical College.

### 4.2 Cell culture and transfection

YES2, KYSE30, KYSE510, KYSE180, KYSE450, KYSE410, and KYSE150 were cultured in RPMI 1640 medium supplemented with 10% FBS and antibiotics. NE2 (esophageal immortalized cell) was kindly provided by Professor Enmin Li from Shantou University and cultured in a 1:1 mixture of EpiLife and dKSFM (Gibco). Cells were incubated at 37°C and 5% CO_2_ in a humidified incubator.

siRNAs targeting circMALAT1 (5′‐UUAGAAACUUUGUCUGCGA‐3′), MSI2 (5′‐CAATGCTGATGTTTGATAA‐3′), and the corresponding negative controls were provided by JTSBIO Co., Ltd (China). pLCDH‐ciR‐circMALAT1 and GV141‐MSI2 plasmid were constructed in Generay (China). pcDNA3.1(+)‐HA‐BTRC plasmid was provided by Geneppl Technology (China). Transfection of the siRNA or plasmid was carried out with Lipofectamine 2000 reagent (Invitrogen, USA) as directed by the manufacturer.

### 4.3 RNA sequencing

CircRNA sequencing was performed to analyze circRNA expression in five pairs of fresh frozen ESCC tissues and para‐carcinoma tissues (RIBOBIO, China). The Beijing Genomics institution in China conducted whole transcriptome sequencing to discover circRNAs that were expressed differently in attached cells and spheroid cells of KYSE450.

### 4.4 RNA extraction, gDNA extraction, PCR, and qPCR

Total RNA of cell lines or fresh clinical specimens were extracted with RNA Express Lysis Buffer (NCM Biotech, China). RNA was isolated from FFPE samples using the RNAprep Pure kit from TIANGEN BIOTECH in China. gDNA of cell lines were extracted with DNA extraction kit (TIANGEN BIOTECH, China). PCR analysis was performed by the 2xTaq Plus MasterMix (Dye) (CWBIO, China) using agarose gel electrophoresis. Total RNA was utilized for cDNA synthesis with Superscript II reverse transcriptase from Invitrogen in the USA. The PerfectStart® Green qPCR SuperMix from TransGen Biotech in China was used for qPCR analysis on the Bio‐Rad System from the USA. Gene expression levels were measured using the 2−ΔΔCt method. Table S6 displays the primer sequences.

### 4.5 Nucleocytoplasmic separation, RNase R, and actinomycin D treatment

RNA from ESCC cells was isolated from nuclear and cytoplasmic fractions using a Nuclear‐Cytosol Extraction Kit (Applygen, China) following the provided guidelines. The RNase R was applied at 37°C using 2 U/µg of RNase R (Geneseed Biotech, China) for a duration of 20 min. To assess mRNA stability, cells were exposed to 100 ng/mL actinomycin D (Sigma–Aldrich, USA) for varying durations of 2, 4, 8, and 12 h following transfection.

### 4.6 Tumor sphere formation assays and spheroids reattached assays

The tumor sphere assay was performed under conditions of low serum levels, low adhesion rates, and low cell density. Following digestion of ESCC cells, 5000 cells were seeded in each well of a low‐adherence six‐well plate containing DMEM/F12 medium (Gibco) with 20 ng/mL EGF, 20 ng/mL bFGF, and 2% B27. Following incubation in spheroid medium for 10–14 days, the quantity and dimensions of cell spheroids were examined using a microscope.

*For tumor sphere reattaching assay*: After culturing in spheroid medium for 7 days, the spheroids were digested and seeded into six‐well plates. CircMALAT1 levels were detected by reattaching the cultured cells to the six‐well plates after 24 h.

### 4.7 Flow cytometry analysis and fluorescence‑activated cell sorting

The cells were dissociated with 0.05% trypsin and EDTA for cell‐surface analysis. 5 × 10^5^ cells were suspended in 100 µL of PBS containing 0.1% BSA, treated with antibodies at the suggested levels, chilled at 4°C for 30 min in darkness, and analyzed using flow cytometry from BD Biosciences in the USA. FlowJo was utilized for the analysis of the data. The flow cytometry test utilized the subsequent antibodies: PE anti‐human CD133 antibody, PE Mouse IgG1, κ isotype Control, FITC anti‐human CD24 antibody, FITC IgG2a, κ Isotype Control (BioLegend, USA), APC anti‐human CD44, APC IgG2b Isotype Control (Proteintech, USA).

### 4.8 Cell proliferation assay

The ESCC cells' capacity for growth was assessed with the xCELLigence Real‐Time Cell Analyzer (RTCA)‐MP system from Acea Biosciences/Roche Applied Science, as detailed in a previous study.^51^ The platform has the capability to monitor the real‐time status of cell growth. Following transfection for 24 h, 2000 transfected cells were then placed in an E‐Plate 96 from Roche Applied Science. The cell index was automatically read every 15 min and displayed as the cell index plus or minus the standard error of the mean.

### 4.9 Wound healing assay

After 24 h of transfection, cells were placed in six‐well dishes. At a cell density of 80–90%, a scratch was created in the center of the well's monolayer using a 200 µL pipette tip. Wound healing within the same scraped line was then observed under a microscope and photographs were taken at the indicated intervals (0 and 24 h).

### 4.10 Colony formation assay

Following transfection for 24 h, 1 × 10^3^ transfected cells were seeded into six‐well plates. Following a period of 10–14 days, the culture plates were rinsed with PBS and then treated with methanol for 10 min to fix them. Afterward, the sample was treated with crystal violet dye for a duration of 10 min. Colonies were examined and measured using a gel imaging analysis system from SYNGENE in the USA.

### 4.11 Transwell assay

Migration and invasion assays were conducted with the Transwell platform from Costar. Following transfection for 24 h, the transfected cells were deprived of serum for 12 h. To conduct the migration assay, 100,000 starved cells were placed in small chambers containing a serum‐free medium and incubated for 12 h, while 800 µL of medium with 20% FBS was added to the bottom wells. Similarly, in the invasion experiment, cell culture inserts were prepared with Matrigel™ Matrix (Corning, USA) following the instructions provided by the manufacturer. A total of 100,000 deprived cells were placed in Matrigel‐coated chambers with serum‐free medium and incubated for 16 h, while 800 µL of medium with 20% FBS was added to the lower wells. The migrating and invading cells were treated with methanol and then stained using crystal violet solution.

### 4.12 Protein extraction and western blot analysis

Cells were lysed with RIPA Lysis Buffer (Applygen) containing cocktail (New Cell & Molecular Biotech, China) and then separated by SDS‐PAGE gels (New Cell & Molecular Biotech). Following gel electrophoresis, proteins were transferred to polyvinylidene fluoride membrane (Millipore, USA) with NcmBlot Rapid Transfer Buffer (New Cell & Molecular Biotech). The membrane was blocked with 5% nonfat milk at room temperature for 30 min, and then was incubated with a specific primary antibody at 4°C overnight. After incubation with specific primary antibody, the membrane was washed and incubated with a secondary antibody (Proteintech) for 1 h. Protein densitometry was quantified by ImageJ software. All antibodies were diluted with NCM Universal Antibody Diluent (New Cell & Molecular Biotech). The assays were performed using the following antibodies: anti‐MSI2 (Proteintech), anti‐β‐actin (Sigma, USA), anti‐cleaved‐caspase3, anti‐cleaved‐caspase8, anti‐cleaved‐caspase9 (Cell Signaling Technology, USA), anti‐NANOG, anti‐OCT4 and anti‐SOX2 (BIOSS, China), anti‐c‐Myc (Santa Cruz Biotechnology, USA).

### 4.13 RNA immunoprecipitation

The interaction between circMALAT1 and MSI2 was assessed using the Magnetic Bead RNA Immunoprecipitation kit from Sigma, following the provided instructions. Magnetic beads were briefly mixed with anti‐MSI2 or IgG, followed by incubation with cell lysis. Following this, RNA was coprecipitated, extracted, and ultimately measured using reverse transcription‐qPCR (RT‐qPCR).

### 4.14 RNA pulldown assays and Mass spectrometry

Biotin‐labeled probes of circMALAT1 (5′‐AGTGTTCGCAGACAAAGTTTCTAAAAATACACCAGCAAAA‐3′) and control (5′‐TTTTGCTGGTGTATTTTTAGAAACTTTGTCTGCGAACACT‐3′) sequences were synthesized in vitro (Generay). The probes were dissolved in DNase/RNase‐free water to 100 µM. The probes were incubated with ESCC cell lysates at room temperature for 2 h, after which streptavidin‐coated beads (GE, USA) were added in for an extra incubation at room temperature for 2 h. The beads were rinsed with wash buffer and the precipitates were identified by western blotting. For mass spectrometry, precipitated proteins were separated through SDS‐PAGE, followed by silver stain with Protein Stains K (Sango Biotech, China) and subsequently sent to Novogene (China) for liquid chromatography‐mass spectrometry assay.

### 4.15 Apoptosis assay

Apoptosis was identified by utilizing the Annexin V‐FITC/PI apoptosis assay kit from neobioscience in accordance with the manufacturer's instructions. Following a 24‐h transfection period, cells were exposed to cisplatin (20 µg/mL) for another 24 h. Subsequently, the cells were subjected to apoptosis, followed by digestion, centrifugation, and resuspension in cold PBS. After removing the supernatant, the cell pellet was then treated with Annexin V‐FITC, PI, and binding buffer for 15 min at 4°C in the dark. Following that, flow cytometry (BD Biosciences, USA) was used to identify cell fluorescence. FlowJo software was utilized for the analysis of the data.

### 4.16 Immunohistochemistry

IHC was conducted with PV‐9000 and DAB chromogenic kit from ZSGB‐BIO, China, following the manufacturer's instructions and using antibodies: anti‐MSI2 (Proteintech), anti‐EpCAM (ABclonal, China), anti‐CD90 (BIOSS), anti‐Ki67 (ZSGB‐BIO), anti‐c‐Myc (Santa Cruz Biotechnology).

### 4.17 Caspase activity assays

The activity of caspases was assessed by utilizing the caspase3/7, caspase8, and caspase9 activity kit from Sangon Biotech, China, following the provided guidelines. Following transfection for 24 h, cells were exposed to cisplatin at a concentration of 20 µg/mL for an additional 24 h. The fluorescence emitted by the cells was measured using VICTOR Nivo from PerkinElmer in Waltham, MA, USA.

### 4.18 Fluorescence in situ hybridization

The location of circMALAT1 was identified with Fluorescent In Situ Hybridization Kit (RIBOBIO) according to the manufacturer's protocol. Briefly, KYSE150 and KYSE450 cells were seeded in glass‐bottom dishes and cultured overnight. The cells were fixed in 4% paraformaldehyde. Fixed cells were incubated with biotin‐labeled circMALAT1 probes (5′‐AGTGTTCGCAGACAAAGTTTCTAAAAATACACCAGCAAAA‐3′) and MSI2 antibody (Proteintech) overnight. The biotin‐labeled circMALAT1 in the fixed cells was labeled with anti‐biotin/RBITC secondary antibody (BIOSS) and Alexa Fluor® 488 secondary antibody (ZSGB‐BIO). Images were obtained using laser‐scanning confocal microscopy (Leica, Germany) and analyzed by Photoshop CS4 (Adobe, USA).

### 4.19 CHX chase assay

After transfection for 24 h, cells were treated with 100 µg/mL CHX (Sigma–Aldrich) for 0, 2, 4, 6, 8, and 10 h. MSI2 protein level was determined by Western blotting.

### 4.20 Co‐immunoprecipitation

Cell lysis was performed using 1% NP‐40 buffer with a cocktail from New Cell & Molecular Biotech. The resulting supernatant was then mixed with a targeted IP antibody or a negative control IgG and incubated at 4°C for 4 h. Following this, Protein A/G agarose beads from MedChemExpress (USA) were introduced to capture the antigen–antibody complex overnight at 4°C. Subsequently, the immunoprecipitates bound to protein A/G beads were gathered and examined through Western blot analysis. For immunoblotting (IB) and IP, the primary antibodies included anti‐MSI2, anti‐BTRC (Proteintech), anti‐β‐actin, anti‐Flag‐tag (Sigma), anti‐HA‐tag (BIOSS), and antiubiquitin (Santa Cruz Biotechnology).

### 4.21 Retroviral infection

The cells were infected following the guidelines provided by the manufacturer, GeneChem (China). The lentivirus of Lenti‐circMALAT1 and Lenti‐Vector were added separately into KYSE150 cells. Cells were infected for 24 h, followed by treatment with puromycin (2 µg/mL) for 7 days to select stable cells, and the infection efficiency was assessed using qPCR. The stable cells were cultured with puromycin (1 µg/mL).

### 4.22 Animal studies

LDA was used to quantify CSC self‐renewal capability and tumorigenicity. Four‐week‐old male NOD/SCID mice were provided by Vital River (China). First, KYSE150 cells stably and highly expressing circMALAT1 (KYSE150‐Lenti‐circMALAT1) and their control cells (KYSE150‐Lenti‐Vector) were cultured in a low‐adherence six‐well plate. After about 7–10 days, the CSC‐like spheroids formed in each well were collected. The spheroids in the cell were separated into individual cells and then gradually reduced to the required amounts. Cells were injected into the armpits of NOD/SCID mice in each group (*n* = 5) at a concentration gradient of 5 × 10^3^, 1 × 10^4^, 5 × 10^4^, and 1 × 10^5^ cells, respectively. The number of tumors was counted after two months.

Male BALB/c nude mice, aged 4 weeks, were acquired from Vital River for the purpose of studying tumorigenicity and lung metastasis in vivo. To established the subcutaneous tumor model, 5 × 10^5^ KYSE150 cells were injected into the upper backs of mice (*n* = 7). The growth of xenografted tumors was observed, and the mice were sacrificed 4 weeks after inoculation. To established the lung metastases model, 1 × 10^6^ KYSE150 cells were injected into the tail veins of 10 nude mice. After a month, the mice were sacrificed and the number of lung metastases in each group was counted. After paraffin embedding, they were sectioned for subsequent research.

### 4.23 Statistical analysis

Each experiment was conducted a minimum of three times. Each experiment was at least three replicas. Data analysis was conducted with SPSS 17.0 and GraphPad 6.0 on a Windows platform. Survival analysis of patients with high and low circMALAT1 expression was conducted using Kaplan–Meier curves and log‐rank tests. When defining high or low expression groups, median expression of circMALAT1 were used as cut‐off points. The Pearson correlation coefficient was utilized to assess the linear relationship between two sets of variables. For all other hypothesis testing, an unpaired two‐tailed Student's *t*‐test was utilized. Statistical significance was defined as *p* < 0.05 in our research.

## AUTHOR CONTRIBUTIONS

Yongmei Song and Zhixu He conceived and designed the experiments. Zitong Zhao, Yingni Deng, and Jing Han completed the experiments. Zitong Zhao and Yingni Deng wrote the manuscript. Liying Ma, Hua Zhang, and Yumeng Zhu analyzed the data and results. Yongmei Song supervised the project and revised the manuscript. The final version of the manuscript was read and approved by all of the authors.

## CONFLICT OF INTEREST STATEMENT

The authors disclose no conflict of interest.

## ETHICS STATEMENT

*For patients*: This study was approved by the Cancer Hospital, Chinese Academy of Medical Sciences, National GCP Center for Anticancer Drugs, The Independent Ethics Committee on March 8, 2021, under the number NCC2021C‐079. The study was conducted in accordance with the Declaration of Helsinki. All human samples used in experiments were approval to collect specimens by the hospital. Informed consent to participate in this study was obtained from all patients.

*For animals*: This study was approved by the Cancer Hospital, Chinese Academy of Medical Sciences, Experimental Animal Ethics Committee and followed Institutional Animal Welfare Guidelines on March 5 2021, under the number NCC2021A111.

## Supporting information

Supporting Information

## Data Availability

The datasets used and analyzed during the current study are available from the corresponding author on reasonable request. The raw transcriptome data have been deposited to the Genome Sequence Archive. GSA accession numbers: HRA007183.
