# Supplementary material for: CircMALAT1 promotes cancer stem‐like properties and chemoresistance via regulating Musashi‐2/c‐Myc axis in esophageal squamous cell carcinoma
Source: MedComm (2020). 2024 Jun 14;5(6):e612. doi: 10.1002/mco2.612 (PMC11176741; doi:10.1002/mco2.612)
Supplement: Supplementary file 1 — Supporting Information [file MCO2-5-e612-s001.docx]

**Supplemental Material for**

**CircMALAT1 promotes cancer stem-like properties and chemoresistance via regulating MSI2/c-Myc axis in esophageal squamous cell carcinoma**

Zitong Zhao^1#^, Yingni Deng^2#^, Jing Han^3#^, Liying Ma^1^, Yumeng Zhu^1,5^, Hua Zhang^4^, Zhixu He^2*^, Yongmei Song^1*^

^1^ Key Laboratory of Cancer and Microbiome, State Key Laboratory of Molecular Oncology, National Cancer Center/National Clinical Research Center for Cancer/Cancer Hospital, Chinese Academy of Medical Sciences and Peking Union Medical College, Beijing, China.

^2^ Collaborative Innovation Center of Tissue Damage Repair and Regeneration Medicine, Zunyi Medical University, Zunyi, China.

^3^Department of Oncology, The Fourth Hospital of Hebei Medical University, Shijiazhuang, Hebei, China.

^4^School of continuing education, Chinese academy of medical sciences & Peking union medical college, Beijing, China.

^5^Beijing No.4 High School International Campus, Beijing, China.

These authors contributed equally: Zitong Zhao, Yingni Deng, Jing Han

*** Correspondence to:**Professor Yongmei Song, State Key Laboratory of Molecular Oncology, National Cancer Center/National Clinical Research Center for Cancer/Cancer Hospital, Chinese Academy of Medical Sciences and Peking Union Medical College, Beijing, China; symlh2006@163.com;

Professor Zhixu He, Collaborative Innovation Center of Tissue Damage Repair and Regeneration Medicine, Zunyi Medical University, Zunyi, China; hzx@gmc.edu.cn.

**This file includes:**

Figures. S1 to S10

Tables. S1 to S6


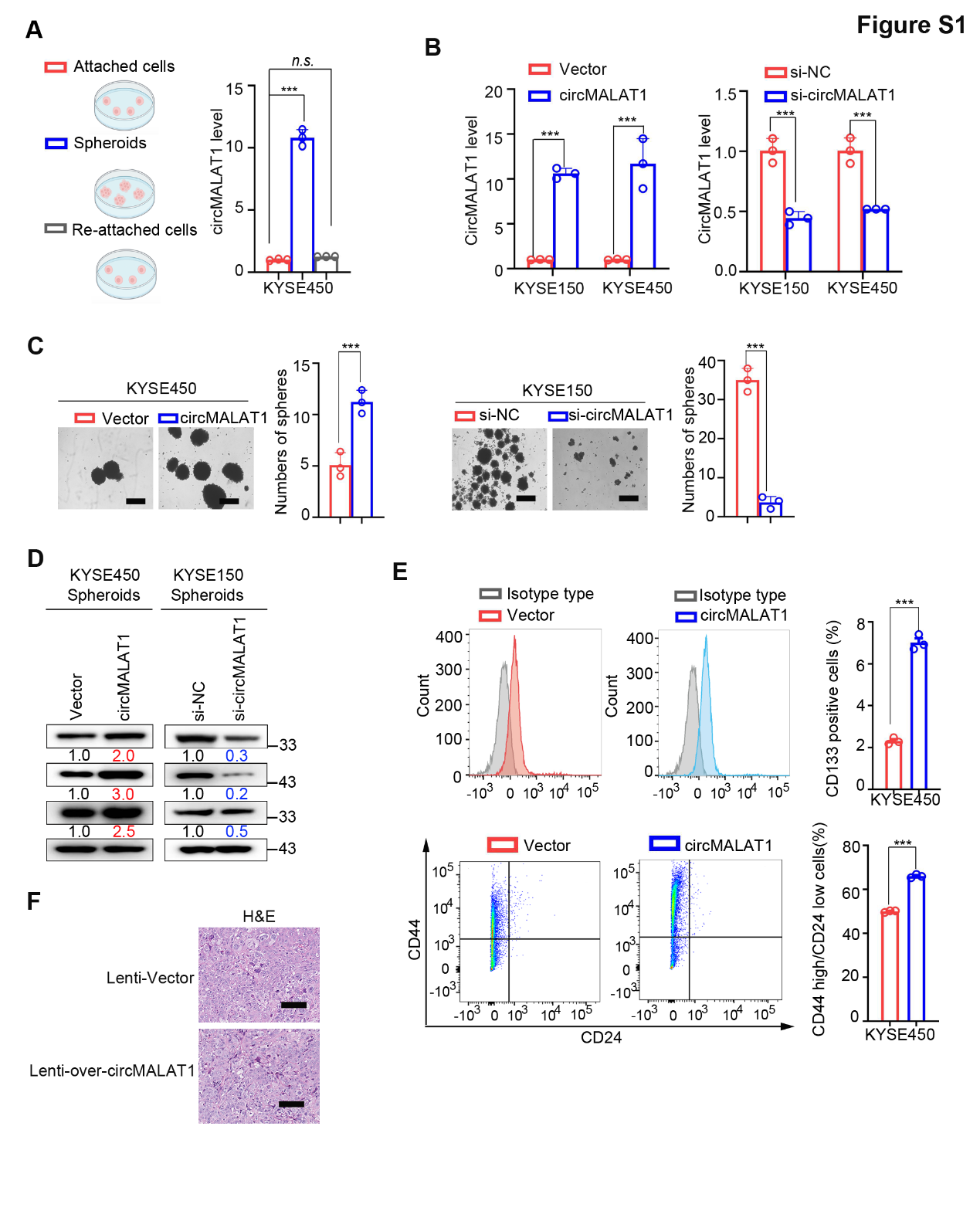


**Figure S1.**

**A** Expression of circMALAT1 in ESCC attached cells, spheroids and spheroids-reattached cells was examined by qPCR.

**B** qRT-PCR analysis of the circMALAT1 levels. KYSE150/KYSE450 cells were transfected with the circMALAT1 or vector. KYSE150/KYSE450 cells were transfected with the siRNA for circMALAT1 or negative control.

**C** Representative images of ESCC spheroids generated from KYSE450 overexpressing or KYSE150 knockdown of circMALAT1 and corresponding control cells. Scale bar, 500 μm.

**D** Western blot analysis of the stemness-associated transcription factors (SOX2, OCT4, Nanog) in spheroids generated from KYSE450 overexpressing or KYSE150 knockdown of circMALAT1 and corresponding control cells.

**E** Flow cytometric analysis of the CD133 and CD44high/CD24low expression level in spheroids generated from KYSE450 overexpressing circMALAT1 and corresponding control cells.

**F** Representative images of immunohistochemical staining of H&E in xenografted tumors. Scale bar, 100 μm.

The data are presented as the mean ± SD, ***P*< 0.01, ****P* < 0.001.


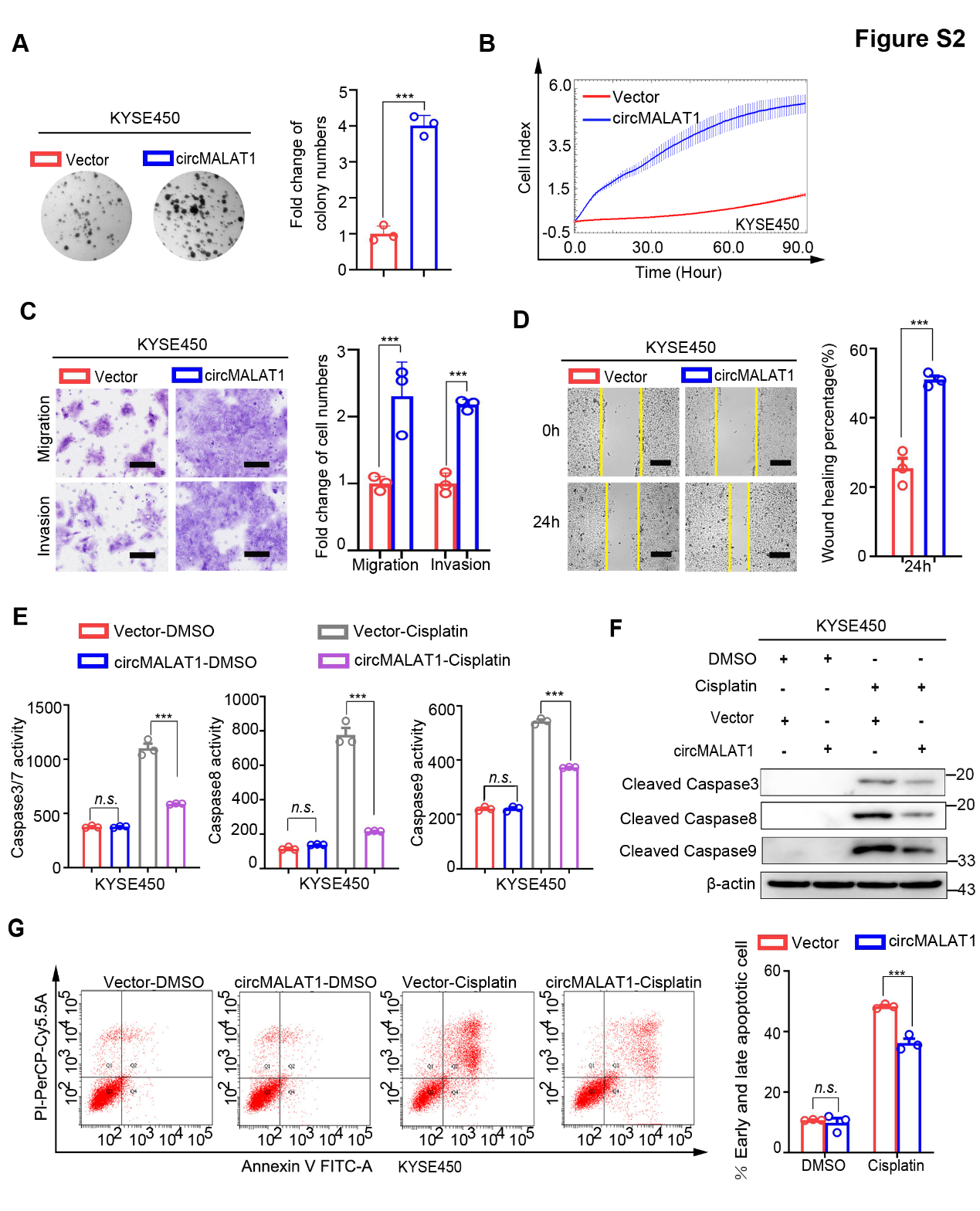


**Figure S2.**

The KYSE450 cells were transfected with circMALAT1 plasmid and corresponding controls.

**A** Cell proliferation ability was evaluated by colony formation.

**B** Growth ability analysis with the xCELLigence Real-Time Cell Analyzer (RTCA)-MP system.

**C** Transwell assays were used to assess the migration and invasion abilities of ESCC cells. Scale bar, 100 μm.

**D** Wound-healing assays were performed to measure the migration abilities of ESCC cells. Scale bar, 200 μm.

**E** Caspase3/7, caspase8 and caspase9 activity was assessed using the fluorogenic substrate after the indicated cells were treated with cisplatin (20 μg/ml) for 24 h.

**F** Western blot analysis of apoptosis-related protein levels after the indicated cells were treated with cisplatin (20 μg/ml) for 24 h.

**G** Flow cytometry analysis of apoptosis (Annexin V/PI) cells after the indicated cells were treated with cisplatin (20 μg/ml) for 24 h. The results are expressed as percentages of the total cells.

The data are presented as the mean ± SD, **P*< 0.05, ****P* < 0.001.


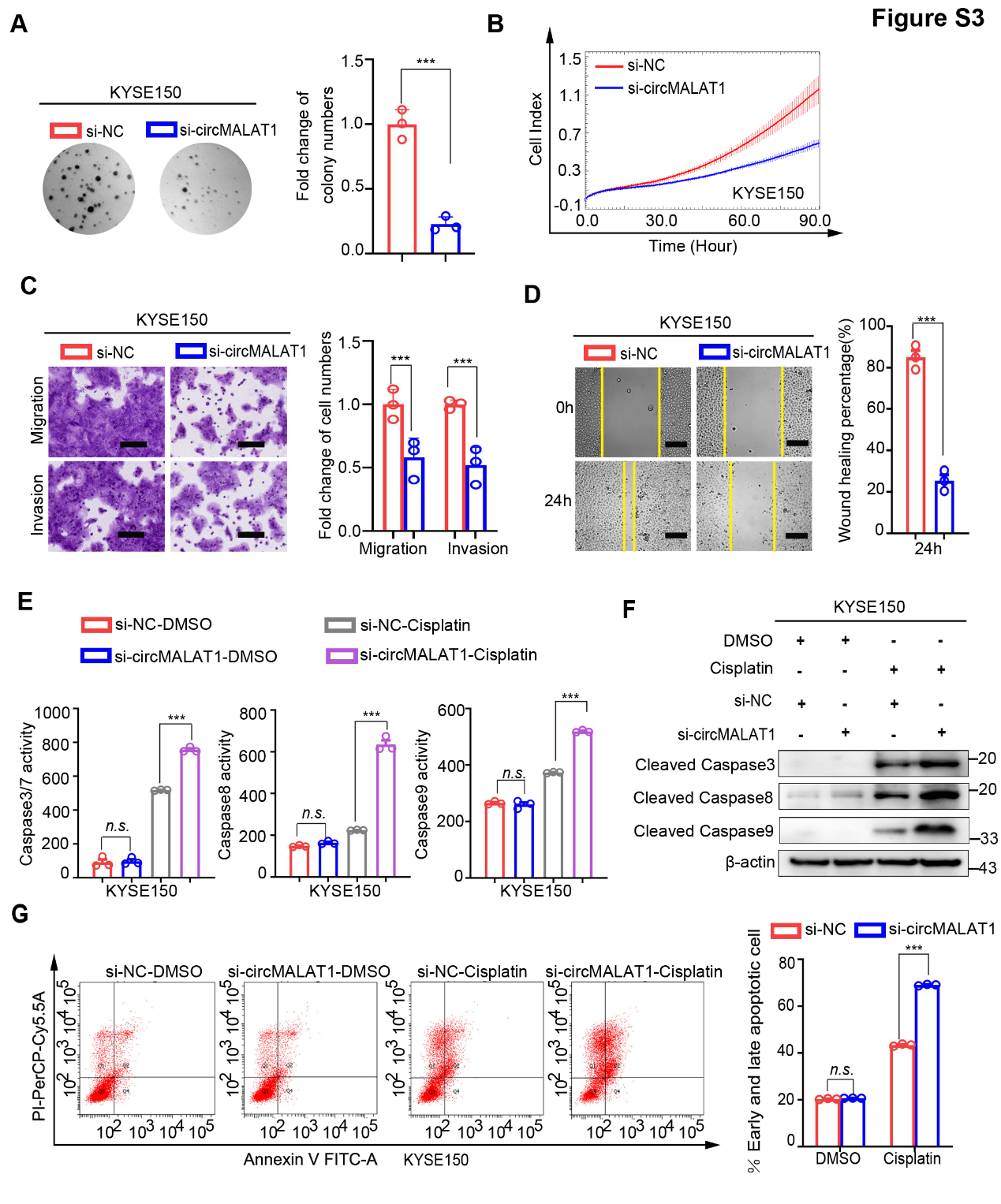


**Figure S3.**

The KYSE150 cells were transfected with circMALAT1-specific siRNA and corresponding controls.

**A** Cell proliferation ability was evaluated by colony formation.

**B** Growth ability analysis with the xCELLigence Real-Time Cell Analyzer (RTCA)-MP system.

**C** Transwell assays were used to measure the migration and invasion abilities of ESCC cells. Scale bar, 100 μm.

**D** Wound-healing assays were performed to assess the migration abilities of ESCC cells. Scale bar, 200 μm.

**E** Caspase3/7, caspase8 and caspase9 activity was assessed using the fluorogenic substrate after the indicated cells were treated with cisplatin (20 μg/ml) for 24 h.

**F** Western blot analysis of apoptosis-related protein levels after the indicated cells were treated with cisplatin (20 μg/ml) for 24 h.

**G** Flow cytometry analysis of apoptosis (Annexin V/PI) cells after the indicated cells were treated with cisplatin (20 μg/ml) for 24 h. The results are expressed as percentages of the total cells.

The data are presented as the mean ± SD, ****P* < 0.001.


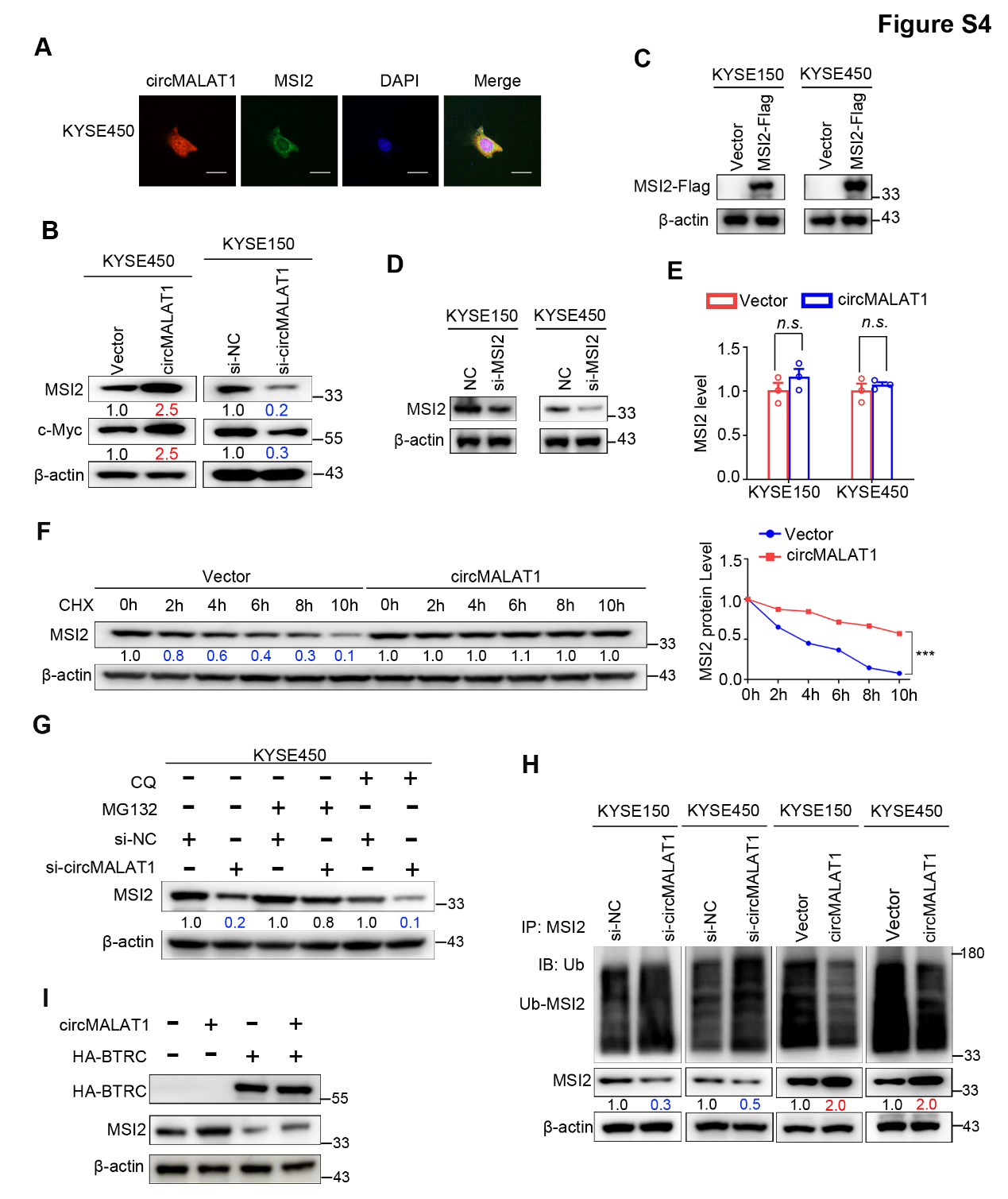


**Figure S4.**

**A** Colocalization analysis of circMALAT1 and MSI2 using protein IF and RNA FISH assays, respectively. Scale bar, 30 μm.

**B** Western blot analysis of the stemness-associated transcription factors (SOX2, OCT4, Nanog) in spheroids generated from KYSE150/KYSE450 overexpressing or knockdown of circMALAT1 and corresponding control cells.

**C-D** Western blot analysis of MSI2 levels.

**E** qPCR analysis of MSI2 in the circMALAT1-overexpressing cells and corresponding control cells.

**F** Western blot detection of MSI2 protein half-life in KYSE150 cells transfected with the circMALAT1 and indicated control and treated with cycloheximide (CHX) (100 μg/ml) for the indicated timepoints.

**G** Western blot of MSI2 levels in the circMALAT1-siRNA cells and corresponding control cells were treated with proteasome inhibitor MG132 (10 μM) or chloroquine (10 μM) for 12 h.

**H** KYSE150 cells were overexpressed/knocked down circMALAT1 and treated with MG132 (10 μM) for 12 h before collection. co-IP was performed with MSI2 antibody in the above-mentioned cells. The polyubiquitinated (Poly-Ub) MSI2 protein was analyzed by Western blot with anti-ubiquitin after co-IP. Ub, ubiquitin.

**I** KYSE150 cells were co-transfected with circMALAT1 and BTRC plasmids, followed by western blotting of the MSI2 and BTRC expression.

**Figure S5-10.** The KYSE150/KYSE450 cells were transfected with circMALAT1 plasmid or co-transfected with si-MSI2. The KYSE150/KYSE450 cells were transfected with si-circMALAT1 or co-transfected with MSI2 plasmid.


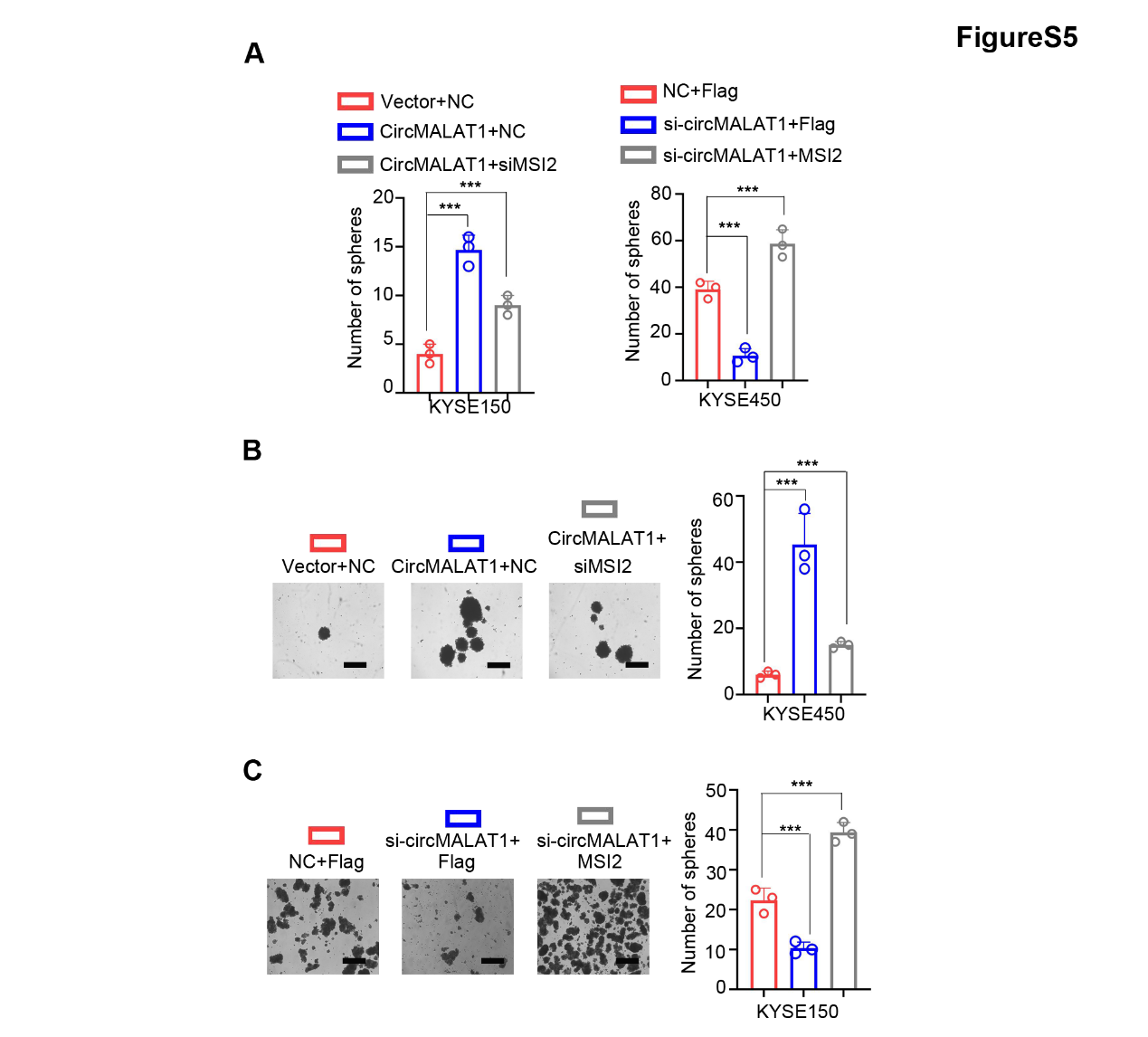


**Figure S5.**

**A** The histograms of number of spheroids in Figure 8A.

**B-C** Representative image of spheroids generated from KYSE150/KYSE450 cells. The number of spheroids was counted and compared. Scale bar, 500 μm.

**
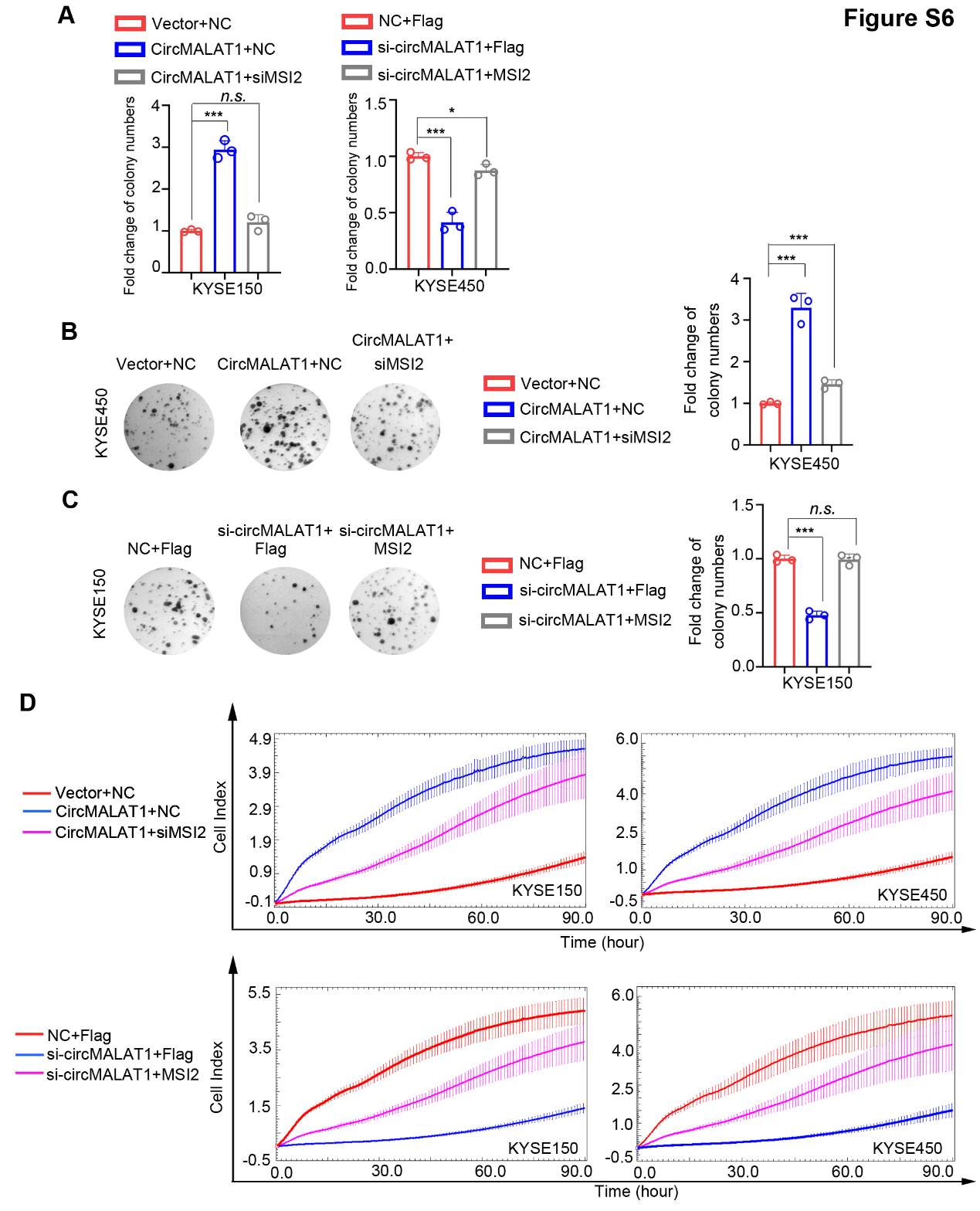
**

**Figure S6.**

**A** The histograms of colony numbers in Figure 8B.

**B-C** Cell proliferation ability was evaluated by colony formation.

**D** Growth ability analysis with the xCELLigence Real-Time Cell Analyzer (RTCA)-MP system.


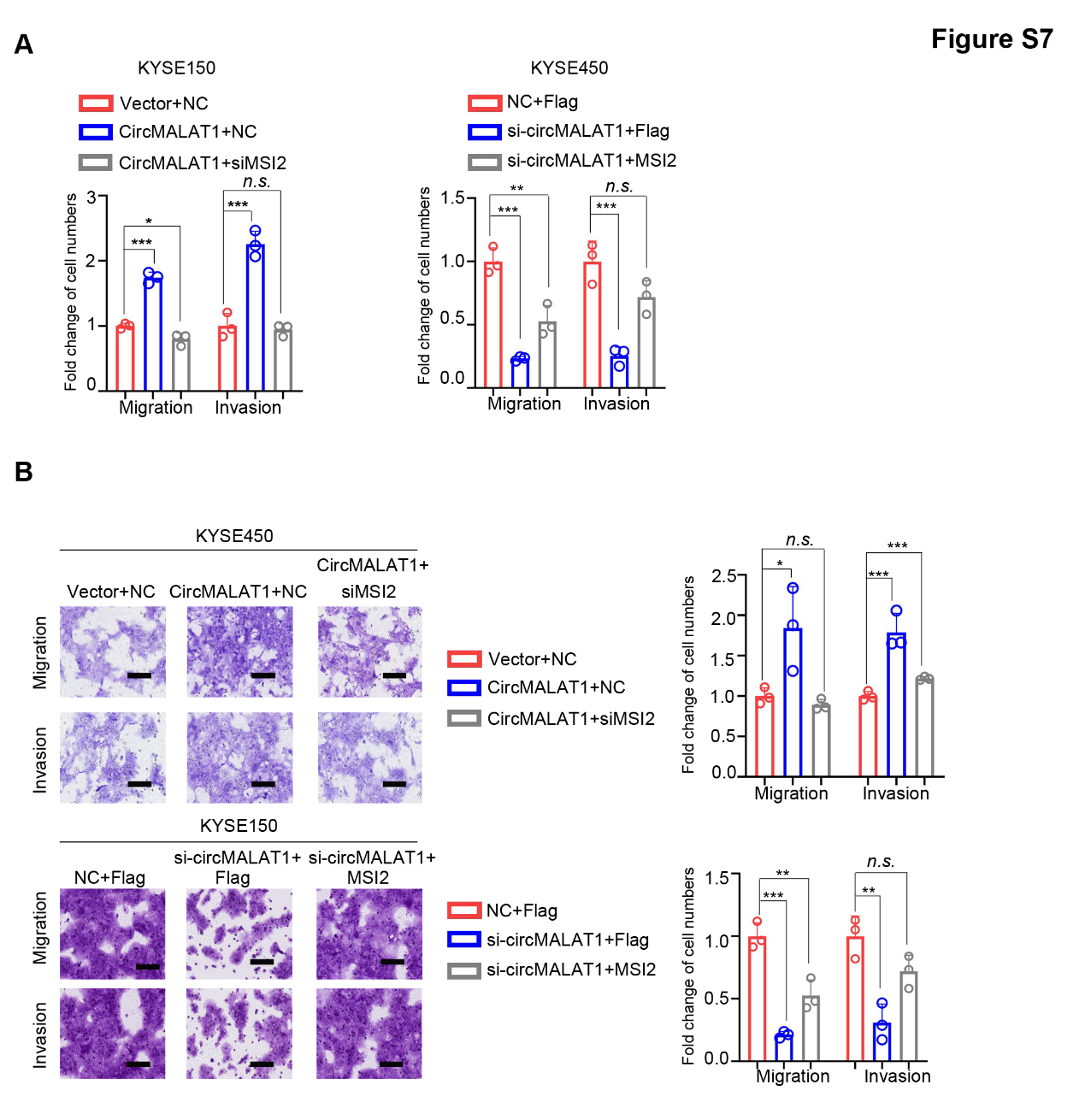


**Figure S7.**

**A** The histograms of Transwell assays in Figure 8C.

**B** Transwell assays were used to measure the migration and invasion abilities of ESCC cells. Scale bar, 100 μm.


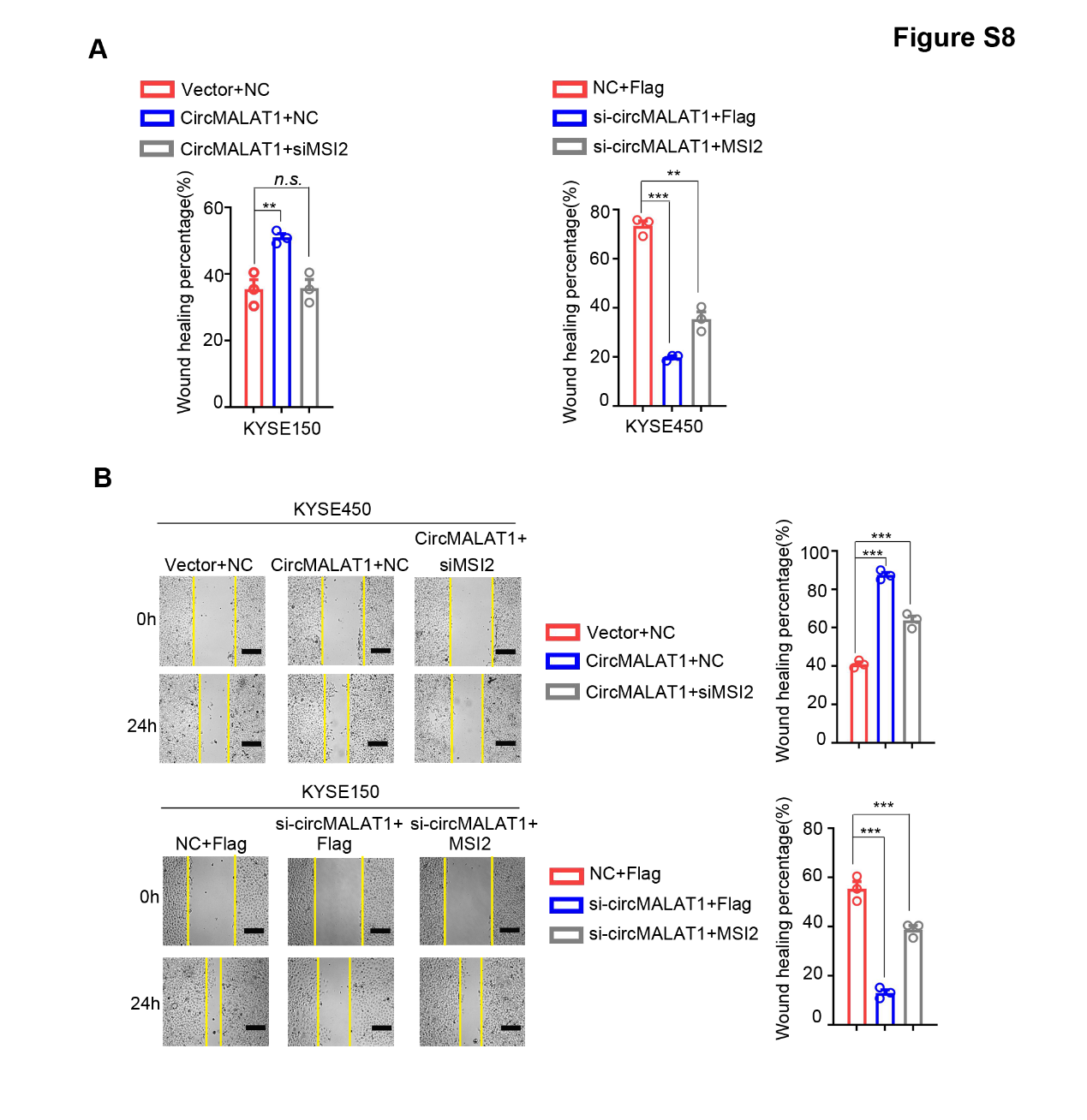


**Figure S8.**

**A** The histograms of Wound-healing assays in Figure 8D.

**B** Wound-healing assays were performed to assess the migration abilities of ESCC cells. Scale bar, 200 μm.


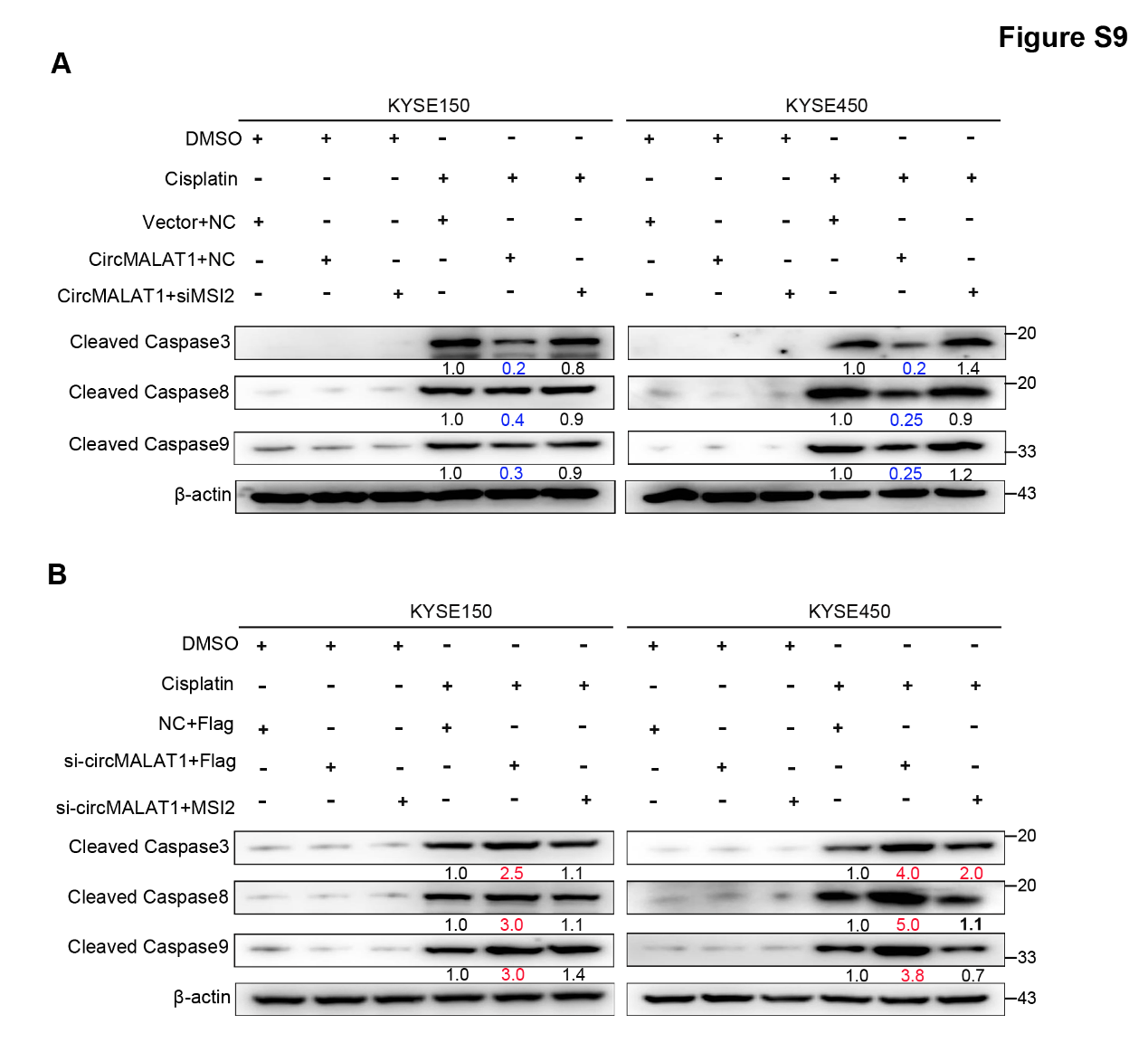


**Figure S9.**

Western blot analysis of apoptosis-related protein levels after the indicated cells were treated with cisplatin (20 μg/ml) for 24 h.


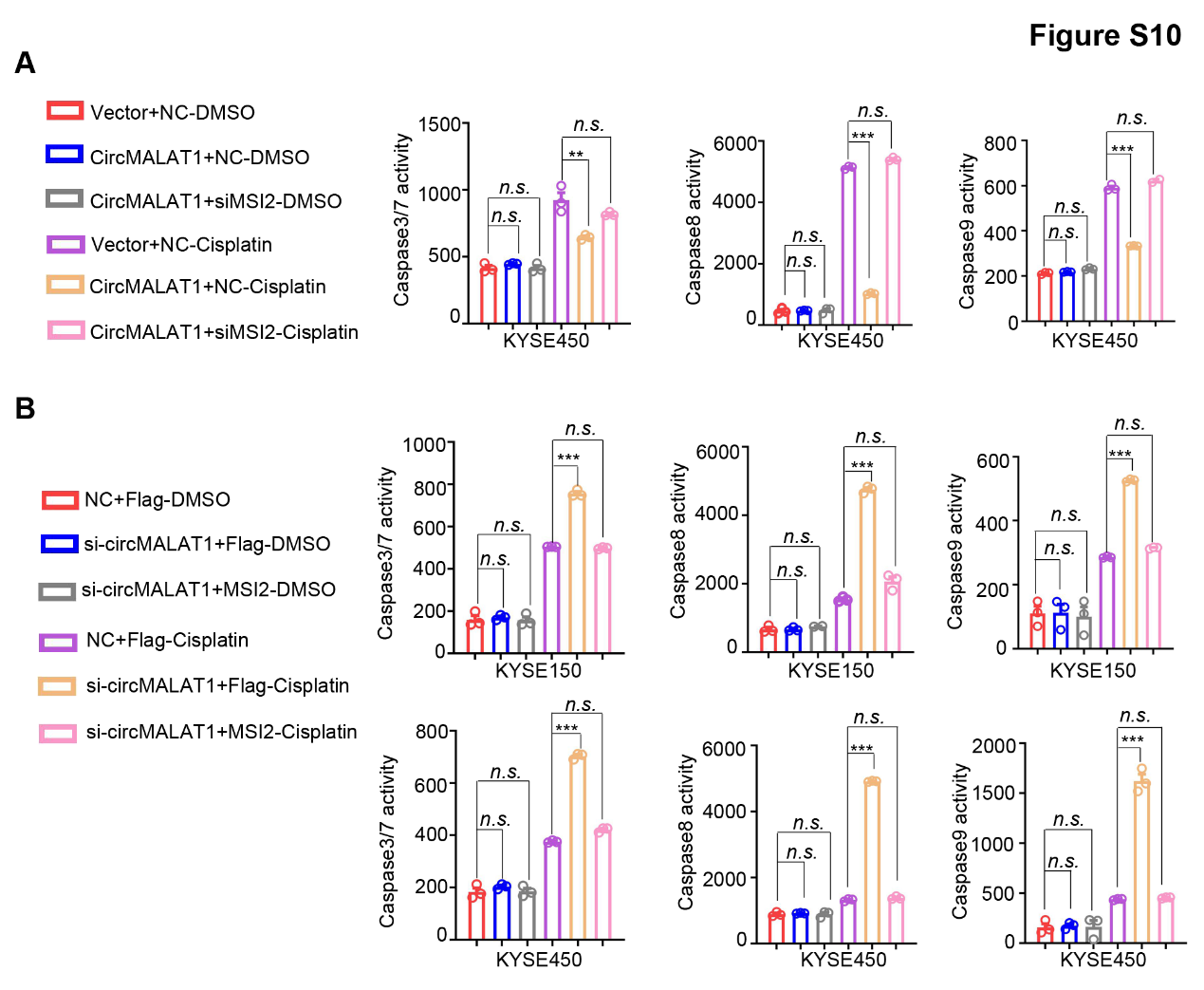


**Figure S10.**

Caspase3/7, caspase8 and caspase9 activity was assessed using the fluorogenic substrate after the indicated cells were treated with cisplatin (20 μg/ml) for 24 h.

The data are presented as the mean ± SD, **P*< 0.05, ***P* < 0.01, ****P* < 0.001.

**TableS1. List of 136 circRNAs were determined to be highly expressed in the ESCC CSC-like cells(Fold Change≥4.0，P≤0.05).**

| st_rna_id | rna_id | gene_id |
| --- | --- | --- |
| C9606_74169 | hsa_circ_0014221 | hsa_circ_0014221 |
| C9606_128118 | hsa_circ_0060532 | hsa_circ_0060532 |
| C9606_10792 | hsa_circ_0014222 | hsa_circ_0014222 |
| C9606_76710 | hsa_circ_0043626 | hsa_circ_0043626 |
| C9606_119623 | hsa_circ_0060535 | hsa_circ_0060535 |
| C9606_10428 | hsa_circ_0060537 | hsa_circ_0060537 |
| C9606_132145 | hsa_circ_0043625 | hsa_circ_0043625 |
| C9606_122612 | hsa_circ_0085651 | hsa_circ_0085651 |
| C9606_27329 | hsa_circ_0074531 | hsa_circ_0074531 |
| C9606_28573 | hsa_circ_0020789 | hsa_circ_0020789 |
| C9606_6238 | hsa_circ_0020790 | hsa_circ_0020790 |
| C9606_78861 | hsa_circ_0042560 | hsa_circ_0042560 |
| C9606_51364 | hsa_circ_0042561 | hsa_circ_0042561 |
| C9606_132590 | hsa_circ_0011080 | hsa_circ_0011080 |
| C9606_120159 | hsa_circ_0014213 | hsa_circ_0014213 |
| C9606_25584 | hsa_circ_0096143 | hsa_circ_0096143 |
| C9606_121168 | hsa_circ_0063227 | hsa_circ_0063227 |
| C9606_99555 | hsa_circ_0058221 | hsa_circ_0058221 |
| C9606_76464 | hsa_circ_0047396 | hsa_circ_0047396 |
| C9606_104867 | hsa_circ_0036524 | hsa_circ_0036524 |
| C9606_40367 | hsa_circ_0019310 | hsa_circ_0019310 |
| C9606_132583 | hsa_circ_0033064 | hsa_circ_0033064 |
| C9606_54975 | hsa_circ_0003987 | hsa_circ_0003987 |
| C9606_82374 | hsa_circ_0096134 | hsa_circ_0096134 |
| C9606_34155 | hsa_circ_0049108 | hsa_circ_0049108 |
| C9606_85606 | hsa_circ_0031594 | hsa_circ_0031594 |
| C9606_48167 | hsa_circ_0063228 | hsa_circ_0063228 |
| C9606_120264 | hsa_circ_0060012 | hsa_circ_0060012 |
| C9606_42963 | hsa_circ_0049111 | hsa_circ_0049111 |
| C9606_80373 | hsa_circ_0085653 | hsa_circ_0085653 |
| C9606_52579 | hsa_circ_0087211 | hsa_circ_0087211 |
| C9606_126014 | hsa_circ_0080910 | hsa_circ_0080910 |
| C9606_88759 | hsa_circ_0109019 | hsa_circ_0109019 |
| C9606_60904 | hsa_circ_0100868 | hsa_circ_0100868 |
| C9606_72217 | hsa_circ_0049116 | hsa_circ_0049116 |
| C9606_42765 | hsa_circ_0085655 | hsa_circ_0085655 |
| C9606_36729 | hsa_circ_0019309 | hsa_circ_0019309 |
| C9606_81728 | hsa_circ_0043642 | hsa_circ_0043642 |
| C9606_65867 | hsa_circ_0061363 | hsa_circ_0061363 |
| C9606_98806 | hsa_circ_0093470 | hsa_circ_0093470 |
| C9606_119407 | hsa_circ_0135821 | hsa_circ_0135821 |
| C9606_117381 | hsa_circ_0085656 | hsa_circ_0085656 |
| C9606_61326 | hsa_circ_0085657 | hsa_circ_0085657 |
| C9606_75406 | hsa_circ_0085647 | hsa_circ_0085647 |
| C9606_130983 | hsa_circ_0085646 | hsa_circ_0085646 |
| C9606_69578 | hsa_circ_0135818 | hsa_circ_0135818 |
| C9606_103471 | hsa_circ_0005915 | hsa_circ_0005915 |
| C9606_56038 | hsa_circ_0075462 | hsa_circ_0075462 |
| C9606_75981 | hsa_circ_0096147 | hsa_circ_0096147 |
| C9606_46516 | hsa_circ_0096142 | hsa_circ_0096142 |
| C9606_88699 | hsa_circ_0096144 | hsa_circ_0096144 |
| C9606_11806 | hsa_circ_0013884 | hsa_circ_0013884 |
| C9606_59908 | hsa_circ_0096168 | hsa_circ_0096168 |
| C9606_68127 | hsa_circ_0096145 | hsa_circ_0096145 |
| C9606_7890 | hsa_circ_0075463 | hsa_circ_0075463 |
| C9606_50490 | hsa_circ_0096131 | hsa_circ_0096131 |
| C9606_19491 | hsa_circ_0096157 | hsa_circ_0096157 |
| C9606_50792 | hsa_circ_0004106 | hsa_circ_0004106 |
| C9606_110505 | hsa_circ_0110692 | hsa_circ_0110692 |
| C9606_84643 | hsa_circ_0002364 | hsa_circ_0002364 |
| C9606_67533 | hsa_circ_0096166 | hsa_circ_0096166 |
| C9606_24843 | hsa_circ_0096136 | hsa_circ_0096136 |
| C9606_43776 | hsa_circ_0088732 | hsa_circ_0088732 |
| C9606_30499 | hsa_circ_0072805 | hsa_circ_0072805 |
| C9606_19587 | hsa_circ_0096152 | hsa_circ_0096152 |
| C9606_110351 | hsa_circ_0085513 | hsa_circ_0085513 |
| C9606_49992 | hsa_circ_0005929 | hsa_circ_0005929 |
| C9606_11785 | hsa_circ_0096127 | hsa_circ_0096127 |
| C9606_137852 | hsa_circ_0025994 | hsa_circ_0025994 |
| C9606_55162 | hsa_circ_0043608 | hsa_circ_0043608 |
| C9606_23555 | hsa_circ_0096165 | hsa_circ_0096165 |
| C9606_60803 | hsa_circ_0096161 | hsa_circ_0096161 |
| C9606_44888 | hsa_circ_0008621 | hsa_circ_0008621 |
| C9606_26609 | hsa_circ_0096135 | hsa_circ_0096135 |
| C9606_87925 | hsa_circ_0096163 | hsa_circ_0096163 |
| C9606_3121 | hsa_circ_0004287 | hsa_circ_0004287 |
| C9606_117889 | hsa_circ_0087212 | hsa_circ_0087212 |
| C9606_120281 | hsa_circ_0000325 | hsa_circ_0000325 |
| C9606_18055 | hsa_circ_0043589 | hsa_circ_0043589 |
| C9606_121623 | hsa_circ_0072454 | hsa_circ_0072454 |
| C9606_14862 | hsa_circ_0002082 | hsa_circ_0002082 |
| C9606_117898 | hsa_circ_0096162 | hsa_circ_0096162 |
| C9606_120787 | hsa_circ_0043607 | hsa_circ_0043607 |
| C9606_110783 | hsa_circ_0096172 | hsa_circ_0096172 |
| C9606_28490 | hsa_circ_0043619 | hsa_circ_0043619 |
| C9606_136310 | hsa_circ_0096138 | hsa_circ_0096138 |
| C9606_33173 | hsa_circ_0096151 | hsa_circ_0096151 |
| C9606_111978 | hsa_circ_0096169 | hsa_circ_0096169 |
| C9606_57561 | hsa_circ_0043638 | hsa_circ_0043638 |
| C9606_64674 | hsa_circ_0000324 | hsa_circ_0000324 |
| C9606_98652 | hsa_circ_0076610 | hsa_circ_0076610 |
| C9606_133293 | hsa_circ_0096170 | hsa_circ_0096170 |
| C9606_96646 | hsa_circ_0043621 | hsa_circ_0043621 |
| C9606_98991 | hsa_circ_0096159 | hsa_circ_0096159 |
| C9606_113161 | hsa_circ_0119363 | hsa_circ_0119363 |
| C9606_81987 | hsa_circ_0013979 | hsa_circ_0013979 |
| C9606_9898 | hsa_circ_0043620 | hsa_circ_0043620 |
| C9606_110928 | hsa_circ_0043618 | hsa_circ_0043618 |
| C9606_66662 | hsa_circ_0119366 | hsa_circ_0119366 |
| C9606_115296 | hsa_circ_0057866 | hsa_circ_0057866 |
| C9606_15594 | hsa_circ_0119364 | hsa_circ_0119364 |
| C9606_72729 | hsa_circ_0096137 | hsa_circ_0096137 |
| C9606_116333 | hsa_circ_0096146 | hsa_circ_0096146 |
| C9606_128939 | hsa_circ_0006999 | hsa_circ_0006999 |
| C9606_60871 | hsa_circ_0116065 | hsa_circ_0116065 |
| C9606_50584 | hsa_circ_0043643 | hsa_circ_0043643 |
| C9606_73309 | hsa_circ_0119365 | hsa_circ_0119365 |
| C9606_125481 | hsa_circ_0043634 | hsa_circ_0043634 |
| C9606_87096 | hsa_circ_0052674 | hsa_circ_0052674 |
| C9606_91926 | hsa_circ_0076609 | hsa_circ_0076609 |
| C9606_37091 | hsa_circ_0043644 | hsa_circ_0043644 |
| C9606_3968 | hsa_circ_0076917 | hsa_circ_0076917 |
| C9606_133950 | hsa_circ_0033484 | hsa_circ_0033484 |
| C9606_31737 | hsa_circ_0096154 | hsa_circ_0096154 |
| C9606_129114 | hsa_circ_0039471 | hsa_circ_0039471 |
| C9606_17236 | hsa_circ_0043640 | hsa_circ_0043640 |
| C9606_50241 | hsa_circ_0087214 | hsa_circ_0087214 |
| C9606_64005 | hsa_circ_0048299 | hsa_circ_0048299 |
| C9606_20152 | hsa_circ_0096148 | hsa_circ_0096148 |
| C9606_50858 | hsa_circ_0102135 | hsa_circ_0102135 |
| C9606_111500 | hsa_circ_0096126 | hsa_circ_0096126 |
| C9606_109318 | hsa_circ_0094123 | hsa_circ_0094123 |
| C9606_2019 | hsa_circ_0043632 | hsa_circ_0043632 |
| C9606_56198 | hsa_circ_0133744 | hsa_circ_0133744 |
| C9606_43962 | hsa_circ_0043633 | hsa_circ_0043633 |
| C9606_131690 | hsa_circ_0096153 | hsa_circ_0096153 |
| C9606_49559 | hsa_circ_0120382 | hsa_circ_0120382 |
| C9606_75817 | hsa_circ_0020527 | hsa_circ_0020527 |
| C9606_118332 | hsa_circ_0096149 | hsa_circ_0096149 |
| C9606_55697 | hsa_circ_0096125 | hsa_circ_0096125 |
| C9606_32863 | hsa_circ_0136209 | hsa_circ_0136209 |
| C9606_43501 | hsa_circ_0031945 | hsa_circ_0031945 |
| C9606_118792 | hsa_circ_0139850 | hsa_circ_0139850 |
| C9606_31820 | hsa_circ_0083744 | hsa_circ_0083744 |
| C9606_103035 | hsa_circ_0007185 | hsa_circ_0007185 |
| C9606_100075 | hsa_circ_0040847 | hsa_circ_0040847 |

**TableS2. List of 63 circRNAs were upregulated in ESCC tissues than adjacent tissues(Fold Change≥5.0，P≤0.05).**

| gene | gene symbol |
| --- | --- |
| hsa_circ:chrUn_gl000220:112412-156384 | RNA45S5 |
| hsa_circ:chr14:20811288-20811559 | RPPH1 |
| hsa_circ:chr14:20811233-20811537 | RPPH1 |
| hsa_circ:chr14:20811237-20811559 | RPPH1 |
| hsa_circ:chr14:20811405-20811568 | RPPH1 |
| hsa_circ:chrUn_gl000220:52120-118788 | RNA45S5 |
| hsa_circ:chrUn_gl000220:108004-118794 | RNA45S5 |
| hsa_circ:chrUn_gl000220:107475-151384 | RNA45S5 |
| hsa_circ:chrUn_gl000220:106709-150559 | RNA45S5 |
| hsa_circ:chr11:65270592-65272066 | MALAT1 |
| hsa_circ:chr12:52885393-52912816 | KRT6A |
| hsa_circ:chr10:34723795-34724188 | PARD3 |
| hsa_circ:chr21:9825578-9826001 | MIR3648 |
| hsa_circ:chr11:65271200-65272066 | MALAT1 |
| hsa_circ:chr10:34722208-34722393 | PARD3 |
| hsa_circ:chr6:26056054-26056299 | HIST1H1C |
| hsa_circ:chr11:65270588-65272066 | MALAT1 |
| hsa_circ:chr11:65272618-65273445 | MALAT1 |
| hsa_circ:chrM:13474-14867 | * |
| hsa_circ:chr17:39740509-39742827 | KRT14 |
| hsa_circ:chr11:65272750-65273093 | MALAT1 |
| hsa_circ:chr11:2018495-2018991 | H19 |
| hsa_circ:chr2:189867697-189868777 | COL3A1 |
| hsa_circ:chr11:65269053-65272194 | MALAT1 |
| hsa_circ:chr9:35657812-35658017 | RMRP |
| hsa_circ:chr12:52912730-52914173 | KRT5 |
| hsa_circ:chr2:189864011-189864303 | COL3A1 |
| hsa_circ:chr2:89156838-89157186 | * |
| hsa_circ:chrM:5338-11645 | * |
| hsa_circ:chr6:58776544-58779091 | * |
| hsa_circ:chr1:153330797-153333291 | S100A9 |
| hsa_circ:chr11:65269040-65269273 | MALAT1 |
| hsa_circ:chr11:65268832-65269153 | MALAT1 |
| hsa_circ:chr10:34734051-34734262 | PARD3 |
| hsa_circ:chrM:14056-16359 | * |
| hsa_circ:chr6:26234555-26234767 | HIST1H1D |
| hsa_circ:chr12:52841323-52882257 | KRT6B |
| hsa_circ:chr6:26123715-26124068 | HIST1H2BC |
| hsa_circ:chr12:6619389-6619674 | NCAPD2 |
| hsa_circ:chr11:65272618-65273368 | MALAT1 |
| hsa_circ:chr12:52882177-52886561 | KRT6A |
| hsa_circ:chr12:52910525-52911478 | KRT5 |
| hsa_circ:chr17:39776976-39780440 | KRT17 |
| hsa_circ:chr6:26285415-26285676 | HIST1H4H |
| hsa_circ:chr11:65272393-65273203 | MALAT1 |
| hsa_circ:chr1:8921162-8926386 | ENO1 |
| hsa_circ:chr12:52912762-52913618 | KRT5 |
| hsa_circ:chr11:65269222-65269864 | MALAT1 |
| hsa_circ:chr2:89156825-89156951 | * |
| hsa_circ:chr17:39775756-39777110 | KRT17 |
| hsa_circ:chrUn_gl000220:112451-156893 | RNA45S5 |
| hsa_circ:chr6:121769023-121769216 | GJA1 |
| hsa_circ:chr17:39739803-39740665 | KRT14 |
| hsa_circ:chr6:27833121-27833526 | HIST1H2AL |
| hsa_circ:chr17:48262062-48262423 | COL1A1 |
| hsa_circ:chr12:52911385-52911758 | KRT5 |
| hsa_circ:chrM:986-8685 | * |
| hsa_circ:chr11:65272414-65273133 | MALAT1 |
| hsa_circ:chr17:39777845-39778661 | KRT17 |
| hsa_circ:chrUn_gl000220:112655-156976 | RNA45S5 |
| hsa_circ:chr21:47409522-47422624 | COL6A1 |
| hsa_circ:chr12:52908722-52910519 | KRT5 |
| hsa_circ:chr19:49468677-49470093 | FTL |

| Prognostic factors | circMALAT1 low（%） | circMALAT1 high（%） | P ^a^ |
| --- | --- | --- | --- |
| Gender |  |  | 0.001 |
| Male | 18(47.4) | 5 (13.5) |  |
| Famale | 20(52.6) | 32 (86.5) |  |
| Age |  |  | 0.083 |
| ＜56 (median) | 7(19.4) | 14 (37.8) |  |
| ≥56 (median) | 29(80.6) | 23 (62.2) |  |
| Differentiation |  |  | 0.032 |
| Well/Moderate | 27 (73.0) | 18 (48.6) |  |
| Poor | 10(27.0) | 19(51.4) |  |
| Stage |  |  | 0.017 |
| T3 | 23(62.2) | 32 (86.5) |  |
| T2 | 14(37.8) | 5 (13.5) |  |
| Lymph node metastasis |  |  | 0.001 |
| Yes | 9 (23.7) | 23 (62.2) |  |
| No | 29(76.3) | 14 (37.8) |  |

**TableS3. The association of clinicopathological characteristics with circMALAT1 expression in ESCC patients (cohort 1).**

Two-sided χ2 test for distributions between low and high expression of circMALAT1.

| Prognostic factors | circMALAT1 low（%） | circMALAT1 high（%） | P ^a^ |
| --- | --- | --- | --- |
| Gender |  |  | 0.04 |
| Male | 41(67.2) | 50 (83.3) |  |
| Famale | 20(33.8) | 10 (16.7) |  |
| Age |  |  | 0.055 |
| ＜56 (median) | 27(44.3) | 37 (61.7) |  |
| ≥56 (median) | 34(55.7) | 23 (38.3) |  |
| Differentiation |  |  | 0.16 |
| Well | 19 (31.1) | 12 (20.0) |  |
| Moderate/Poor | 42(68.9) | 48(80.0) |  |
| Lymph node metastasis |  |  | 0.000 |
| Yes | 3 (4.9) | 38 (63.3) |  |
| No | 58(95.1) | 22(36.7) |  |

**Supplementary Table 4. The association of clinicopathological characteristics with circMALAT1 expression in ESCC patients (cohort 2).**

Two-sided χ2 test for distributions between low and high expression of circMALAT1.

**TableS5. List of the potential circMALAT1-interacting proteins identified by LC-MSMS spectrometry after pull-down with the biontin-labeled circMALAT1 probe.**

| Accession | Description |
| --- | --- |
| Q96L58 | Beta-1,3-galactosyltransferase 6 OS=Homo sapiens OX=9606 GN=B3GALT6 PE=1 SV=2 |
| Q12926 | ELAV-like protein 2 OS=Homo sapiens OX=9606 GN=ELAVL2 PE=1 SV=2 |
| Q86SE5 | RNA-binding Raly-like protein OS=Homo sapiens OX=9606 GN=RALYL PE=1 SV=2 |
| Q9P2K5 | Myelin expression factor 2 OS=Homo sapiens OX=9606 GN=MYEF2 PE=1 SV=3 |
| Q9H1C4 | Protein unc-93 homolog B1 OS=Homo sapiens OX=9606 GN=UNC93B1 PE=1 SV=2 |
| Q9H0P0 | Cytosolic 5'-nucleotidase 3A OS=Homo sapiens OX=9606 GN=NT5C3A PE=1 SV=3 |
| P54619 | 5'-AMP-activated protein kinase subunit gamma-1 OS=Homo sapiens OX=9606 GN=PRKAG1 PE=1 SV=1 |
| Q86U06 | Probable RNA-binding protein 23 OS=Homo sapiens OX=9606 GN=RBM23 PE=1 SV=1 |
| Q14576 | ELAV-like protein 3 OS=Homo sapiens OX=9606 GN=ELAVL3 PE=2 SV=3 |
| P08670 | Vimentin OS=Homo sapiens OX=9606 GN=VIM PE=1 SV=4 |
| Q6ZVX7 | F-box only protein 50 OS=Homo sapiens OX=9606 GN=NCCRP1 PE=1 SV=1 |
| Q15397 | Pumilio homolog 3 OS=Homo sapiens OX=9606 GN=PUM3 PE=1 SV=3 |
| O43347 | RNA-binding protein Musashi homolog 1 OS=Homo sapiens OX=9606 GN=MSI1 PE=1 SV=1 |
| O60248 | Protein SOX-15 OS=Homo sapiens OX=9606 GN=SOX15 PE=1 SV=1 |
| Q14997 | Proteasome activator complex subunit 4 OS=Homo sapiens OX=9606 GN=PSME4 PE=1 SV=2 |
| Q6NSI4 | RPA-related protein RADX OS=Homo sapiens OX=9606 GN=RADX PE=1 SV=2 |
| Q9BZZ5 | Apoptosis inhibitor 5 OS=Homo sapiens OX=9606 GN=API5 PE=1 SV=3 |
| Q9H3K2 | Growth hormone-inducible transmembrane protein OS=Homo sapiens OX=9606 GN=GHITM PE=1 SV=2 |
| P30838 | Aldehyde dehydrogenase, dimeric NADP-preferring OS=Homo sapiens OX=9606 GN=ALDH3A1 PE=1 SV=3 |
| Q14318 | Peptidyl-prolyl cis-trans isomerase FKBP8 OS=Homo sapiens OX=9606 GN=FKBP8 PE=1 SV=2 |
| Q8NBS9 | Thioredoxin domain-containing protein 5 OS=Homo sapiens OX=9606 GN=TXNDC5 PE=1 SV=2 |
| Q13769 | THO complex subunit 5 homolog OS=Homo sapiens OX=9606 GN=THOC5 PE=1 SV=2 |
| P20290 | Transcription factor BTF3 OS=Homo sapiens OX=9606 GN=BTF3 PE=1 SV=1 |
| P30533 | Alpha-2-macroglobulin receptor-associated protein OS=Homo sapiens OX=9606 GN=LRPAP1 PE=1 SV=1 |
| Q8WU90 | Zinc finger CCCH domain-containing protein 15 OS=Homo sapiens OX=9606 GN=ZC3H15 PE=1 SV=1 |
| P31937 | 3-hydroxyisobutyrate dehydrogenase, mitochondrial OS=Homo sapiens OX=9606 GN=HIBADH PE=1 SV=2 |
| Q13428 | Treacle protein OS=Homo sapiens OX=9606 GN=TCOF1 PE=1 SV=3 |
| Q9UNZ2 | NSFL1 cofactor p47 OS=Homo sapiens OX=9606 GN=NSFL1C PE=1 SV=2 |
| Q96G03 | Phosphoglucomutase-2 OS=Homo sapiens OX=9606 GN=PGM2 PE=1 SV=4 |
| Q00688 | Peptidyl-prolyl cis-trans isomerase FKBP3 OS=Homo sapiens OX=9606 GN=FKBP3 PE=1 SV=1 |
| Q6UW68 | Transmembrane protein 205 OS=Homo sapiens OX=9606 GN=TMEM205 PE=1 SV=1 |
| P16190 | HLA class I histocompatibility antigen, A-33 alpha chain OS=Homo sapiens OX=9606 GN=HLA-A PE=1 SV=3 |
| Q8TC07 | TBC1 domain family member 15 OS=Homo sapiens OX=9606 GN=TBC1D15 PE=1 SV=2 |
| P46109 | Crk-like protein OS=Homo sapiens OX=9606 GN=CRKL PE=1 SV=1 |
| O43399 | Tumor protein D54 OS=Homo sapiens OX=9606 GN=TPD52L2 PE=1 SV=2 |
| Q9H501 | ESF1 homolog OS=Homo sapiens OX=9606 GN=ESF1 PE=1 SV=1 |
| Q6L8Q7 | 2',5'-phosphodiesterase 12 OS=Homo sapiens OX=9606 GN=PDE12 PE=1 SV=2 |
| Q92879 | CUGBP Elav-like family member 1 OS=Homo sapiens OX=9606 GN=CELF1 PE=1 SV=2 |
| P51610 | Host cell factor 1 OS=Homo sapiens OX=9606 GN=HCFC1 PE=1 SV=2 |
| O95292 | Vesicle-associated membrane protein-associated protein B/C OS=Homo sapiens OX=9606 GN=VAPB PE=1 SV=3 |
| Q05682 | Caldesmon OS=Homo sapiens OX=9606 GN=CALD1 PE=1 SV=3 |
| Q92820 | Gamma-glutamyl hydrolase OS=Homo sapiens OX=9606 GN=GGH PE=1 SV=2 |
| P83111 | Serine beta-lactamase-like protein LACTB, mitochondrial OS=Homo sapiens OX=9606 GN=LACTB PE=1 SV=2 |
| Q16555 | Dihydropyrimidinase-related protein 2 OS=Homo sapiens OX=9606 GN=DPYSL2 PE=1 SV=1 |
| Q8IWA0 | WD repeat-containing protein 75 OS=Homo sapiens OX=9606 GN=WDR75 PE=1 SV=1 |
| Q16851 | UTP--glucose-1-phosphate uridylyltransferase OS=Homo sapiens OX=9606 GN=UGP2 PE=1 SV=5 |
| P26196 | Probable ATP-dependent RNA helicase DDX6 OS=Homo sapiens OX=9606 GN=DDX6 PE=1 SV=2 |
| Q6PKG0 | La-related protein 1 OS=Homo sapiens OX=9606 GN=LARP1 PE=1 SV=2 |
| P49756 | RNA-binding protein 25 OS=Homo sapiens OX=9606 GN=RBM25 PE=1 SV=3 |
| Q13409 | Cytoplasmic dynein 1 intermediate chain 2 OS=Homo sapiens OX=9606 GN=DYNC1I2 PE=1 SV=3 |
| P16422 | Epithelial cell adhesion molecule OS=Homo sapiens OX=9606 GN=EPCAM PE=1 SV=2 |
| P51858 | Hepatoma-derived growth factor OS=Homo sapiens OX=9606 GN=HDGF PE=1 SV=1 |
| P46108 | Adapter molecule crk OS=Homo sapiens OX=9606 GN=CRK PE=1 SV=2 |
| Q53EL6 | Programmed cell death protein 4 OS=Homo sapiens OX=9606 GN=PDCD4 PE=1 SV=2 |
| P55145 | Mesencephalic astrocyte-derived neurotrophic factor OS=Homo sapiens OX=9606 GN=MANF PE=1 SV=3 |
| Q02127 | Dihydroorotate dehydrogenase (quinone), mitochondrial OS=Homo sapiens OX=9606 GN=DHODH PE=1 SV=3 |
| Q13426 | DNA repair protein XRCC4 OS=Homo sapiens OX=9606 GN=XRCC4 PE=1 SV=2 |
| O15173 | Membrane-associated progesterone receptor component 2 OS=Homo sapiens OX=9606 GN=PGRMC2 PE=1 SV=1 |
| P33240 | Cleavage stimulation factor subunit 2 OS=Homo sapiens OX=9606 GN=CSTF2 PE=1 SV=1 |
| P05556 | Integrin beta-1 OS=Homo sapiens OX=9606 GN=ITGB1 PE=1 SV=2 |
| P55209 | Nucleosome assembly protein 1-like 1 OS=Homo sapiens OX=9606 GN=NAP1L1 PE=1 SV=1 |
| Q96A49 | Synapse-associated protein 1 OS=Homo sapiens OX=9606 GN=SYAP1 PE=1 SV=1 |
| Q06323 | Proteasome activator complex subunit 1 OS=Homo sapiens OX=9606 GN=PSME1 PE=1 SV=1 |
| Q9Y3T9 | Nucleolar complex protein 2 homolog OS=Homo sapiens OX=9606 GN=NOC2L PE=1 SV=4 |
| P82675 | 28S ribosomal protein S5, mitochondrial OS=Homo sapiens OX=9606 GN=MRPS5 PE=1 SV=2 |
| Q96M27 | Protein PRRC1 OS=Homo sapiens OX=9606 GN=PRRC1 PE=1 SV=1 |
| Q9Y6D9 | Mitotic spindle assembly checkpoint protein MAD1 OS=Homo sapiens OX=9606 GN=MAD1L1 PE=1 SV=2 |
| Q16740 | ATP-dependent Clp protease proteolytic subunit, mitochondrial OS=Homo sapiens OX=9606 GN=CLPP PE=1 SV=1 |
| Q9Y2W2 | WW domain-binding protein 11 OS=Homo sapiens OX=9606 GN=WBP11 PE=1 SV=1 |
| O95336 | 6-phosphogluconolactonase OS=Homo sapiens OX=9606 GN=PGLS PE=1 SV=2 |
| Q9Y676 | 28S ribosomal protein S18b, mitochondrial OS=Homo sapiens OX=9606 GN=MRPS18B PE=1 SV=1 |
| P82673 | 28S ribosomal protein S35, mitochondrial OS=Homo sapiens OX=9606 GN=MRPS35 PE=1 SV=1 |
| Q9H2U2 | Inorganic pyrophosphatase 2, mitochondrial OS=Homo sapiens OX=9606 GN=PPA2 PE=1 SV=2 |
| P13796 | Plastin-2 OS=Homo sapiens OX=9606 GN=LCP1 PE=1 SV=6 |
| Q16890 | Tumor protein D53 OS=Homo sapiens OX=9606 GN=TPD52L1 PE=1 SV=1 |
| Q8TBC4 | NEDD8-activating enzyme E1 catalytic subunit OS=Homo sapiens OX=9606 GN=UBA3 PE=1 SV=2 |
| Q93034 | Cullin-5 OS=Homo sapiens OX=9606 GN=CUL5 PE=1 SV=4 |
| Q9UJA5 | tRNA (adenine(58)-N(1))-methyltransferase non-catalytic subunit TRM6 OS=Homo sapiens OX=9606 GN=TRMT6 PE=1 SV=1 |
| Q13423 | NAD(P) transhydrogenase, mitochondrial OS=Homo sapiens OX=9606 GN=NNT PE=1 SV=3 |
| Q9UKX7 | Nuclear pore complex protein Nup50 OS=Homo sapiens OX=9606 GN=NUP50 PE=1 SV=2 |
| P26006 | Integrin alpha-3 OS=Homo sapiens OX=9606 GN=ITGA3 PE=1 SV=5 |
| Q9NVH0 | Exonuclease 3'-5' domain-containing protein 2 OS=Homo sapiens OX=9606 GN=EXD2 PE=1 SV=2 |
| O00461 | Golgi integral membrane protein 4 OS=Homo sapiens OX=9606 GN=GOLIM4 PE=1 SV=1 |
| Q9ULW0 | Targeting protein for Xklp2 OS=Homo sapiens OX=9606 GN=TPX2 PE=1 SV=2 |
| Q13505 | Metaxin-1 OS=Homo sapiens OX=9606 GN=MTX1 PE=1 SV=3 |
| P17252 | Protein kinase C alpha type OS=Homo sapiens OX=9606 GN=PRKCA PE=1 SV=4 |
| Q9NT62 | Ubiquitin-like-conjugating enzyme ATG3 OS=Homo sapiens OX=9606 GN=ATG3 PE=1 SV=1 |
| Q9Y3D9 | 28S ribosomal protein S23, mitochondrial OS=Homo sapiens OX=9606 GN=MRPS23 PE=1 SV=2 |
| P51553 | Isocitrate dehydrogenase [NAD] subunit gamma, mitochondrial OS=Homo sapiens OX=9606 GN=IDH3G PE=1 SV=1 |
| Q08477 | Docosahexaenoic acid omega-hydroxylase CYP4F3 OS=Homo sapiens OX=9606 GN=CYP4F3 PE=1 SV=2 |
| Q9NXF1 | Testis-expressed protein 10 OS=Homo sapiens OX=9606 GN=TEX10 PE=1 SV=2 |
| Q9GZZ1 | N-alpha-acetyltransferase 50 OS=Homo sapiens OX=9606 GN=NAA50 PE=1 SV=1 |
| O43172 | U4/U6 small nuclear ribonucleoprotein Prp4 OS=Homo sapiens OX=9606 GN=PRPF4 PE=1 SV=2 |
| Q9BY44 | Eukaryotic translation initiation factor 2A OS=Homo sapiens OX=9606 GN=EIF2A PE=1 SV=3 |
| Q9NQW6 | Anillin OS=Homo sapiens OX=9606 GN=ANLN PE=1 SV=2 |
| Q96ME1 | F-box/LRR-repeat protein 18 OS=Homo sapiens OX=9606 GN=FBXL18 PE=1 SV=2 |
| Q92890 | Ubiquitin recognition factor in ER-associated degradation protein 1 OS=Homo sapiens OX=9606 GN=UFD1 PE=1 SV=3 |
| Q96AB3 | Isochorismatase domain-containing protein 2 OS=Homo sapiens OX=9606 GN=ISOC2 PE=1 SV=1 |
| Q658P3 | Metalloreductase STEAP3 OS=Homo sapiens OX=9606 GN=STEAP3 PE=1 SV=2 |
| P49585 | Choline-phosphate cytidylyltransferase A OS=Homo sapiens OX=9606 GN=PCYT1A PE=1 SV=2 |
| O60493 | Sorting nexin-3 OS=Homo sapiens OX=9606 GN=SNX3 PE=1 SV=3 |
| Q6P9B6 | TLD domain-containing protein 1 OS=Homo sapiens OX=9606 GN=TLDC1 PE=1 SV=2 |
| Q7L2E3 | Putative ATP-dependent RNA helicase DHX30 OS=Homo sapiens OX=9606 GN=DHX30 PE=1 SV=1 |
| Q8NEF9 | Serum response factor-binding protein 1 OS=Homo sapiens OX=9606 GN=SRFBP1 PE=1 SV=1 |
| Q8TDX7 | Serine/threonine-protein kinase Nek7 OS=Homo sapiens OX=9606 GN=NEK7 PE=1 SV=1 |
| P78316 | Nucleolar protein 14 OS=Homo sapiens OX=9606 GN=NOP14 PE=1 SV=3 |
| Q13586 | Stromal interaction molecule 1 OS=Homo sapiens OX=9606 GN=STIM1 PE=1 SV=3 |
| Q8IUE6 | Histone H2A type 2-B OS=Homo sapiens OX=9606 GN=HIST2H2AB PE=1 SV=3 |
| Q9BSJ2 | Gamma-tubulin complex component 2 OS=Homo sapiens OX=9606 GN=TUBGCP2 PE=1 SV=2 |
| O00515 | Ladinin-1 OS=Homo sapiens OX=9606 GN=LAD1 PE=1 SV=2 |
| Q15018 | BRISC complex subunit Abraxas 2 OS=Homo sapiens OX=9606 GN=ABRAXAS2 PE=1 SV=2 |
| Q9Y3B3 | Transmembrane emp24 domain-containing protein 7 OS=Homo sapiens OX=9606 GN=TMED7 PE=1 SV=2 |
| Q9UHX1 | Poly(U)-binding-splicing factor PUF60 OS=Homo sapiens OX=9606 GN=PUF60 PE=1 SV=1 |
| Q9NZ45 | CDGSH iron-sulfur domain-containing protein 1 OS=Homo sapiens OX=9606 GN=CISD1 PE=1 SV=1 |
| Q15796 | Mothers against decapentaplegic homolog 2 OS=Homo sapiens OX=9606 GN=SMAD2 PE=1 SV=1 |
| Q9BRJ2 | 39S ribosomal protein L45, mitochondrial OS=Homo sapiens OX=9606 GN=MRPL45 PE=1 SV=2 |
| Q9Y3B7 | 39S ribosomal protein L11, mitochondrial OS=Homo sapiens OX=9606 GN=MRPL11 PE=1 SV=1 |
| O75695 | Protein XRP2 OS=Homo sapiens OX=9606 GN=RP2 PE=1 SV=4 |
| Q9Y3B9 | RRP15-like protein OS=Homo sapiens OX=9606 GN=RRP15 PE=1 SV=2 |
| P14550 | Alcohol dehydrogenase [NADP(+)] OS=Homo sapiens OX=9606 GN=AKR1A1 PE=1 SV=3 |
| Q92973 | Transportin-1 OS=Homo sapiens OX=9606 GN=TNPO1 PE=1 SV=2 |
| O43815 | Striatin OS=Homo sapiens OX=9606 GN=STRN PE=1 SV=4 |
| O75607 | Nucleoplasmin-3 OS=Homo sapiens OX=9606 GN=NPM3 PE=1 SV=3 |
| Q9Y5J7 | Mitochondrial import inner membrane translocase subunit Tim9 OS=Homo sapiens OX=9606 GN=TIMM9 PE=1 SV=1 |
| Q16270 | Insulin-like growth factor-binding protein 7 OS=Homo sapiens OX=9606 GN=IGFBP7 PE=1 SV=1 |
| Q8TCC3 | 39S ribosomal protein L30, mitochondrial OS=Homo sapiens OX=9606 GN=MRPL30 PE=1 SV=1 |
| Q9Y4E1 | WASH complex subunit 2C OS=Homo sapiens OX=9606 GN=WASHC2C PE=1 SV=3 |
| P23381 | Tryptophan--tRNA ligase, cytoplasmic OS=Homo sapiens OX=9606 GN=WARS PE=1 SV=2 |
| O14936 | Peripheral plasma membrane protein CASK OS=Homo sapiens OX=9606 GN=CASK PE=1 SV=3 |
| O95363 | Phenylalanine--tRNA ligase, mitochondrial OS=Homo sapiens OX=9606 GN=FARS2 PE=1 SV=1 |
| O75223 | Gamma-glutamylcyclotransferase OS=Homo sapiens OX=9606 GN=GGCT PE=1 SV=1 |
| O94766 | Galactosylgalactosylxylosylprotein 3-beta-glucuronosyltransferase 3 OS=Homo sapiens OX=9606 GN=B3GAT3 PE=1 SV=2 |
| P16401 | Histone H1.5 OS=Homo sapiens OX=9606 GN=HIST1H1B PE=1 SV=3 |
| Q9BZF1 | Oxysterol-binding protein-related protein 8 OS=Homo sapiens OX=9606 GN=OSBPL8 PE=1 SV=3 |
| Q86WR7 | Proline and serine-rich protein 2 OS=Homo sapiens OX=9606 GN=PROSER2 PE=1 SV=2 |
| Q12765 | Secernin-1 OS=Homo sapiens OX=9606 GN=SCRN1 PE=1 SV=2 |
| Q9H6F5 | Coiled-coil domain-containing protein 86 OS=Homo sapiens OX=9606 GN=CCDC86 PE=1 SV=1 |
| Q96IJ6 | Mannose-1-phosphate guanyltransferase alpha OS=Homo sapiens OX=9606 GN=GMPPA PE=1 SV=1 |
| Q5BKZ1 | DBIRD complex subunit ZNF326 OS=Homo sapiens OX=9606 GN=ZNF326 PE=1 SV=2 |
| P29590 | Protein PML OS=Homo sapiens OX=9606 GN=PML PE=1 SV=3 |
| P08962 | CD63 antigen OS=Homo sapiens OX=9606 GN=CD63 PE=1 SV=2 |
| Q15287 | RNA-binding protein with serine-rich domain 1 OS=Homo sapiens OX=9606 GN=RNPS1 PE=1 SV=1 |
| Q96DB5 | Regulator of microtubule dynamics protein 1 OS=Homo sapiens OX=9606 GN=RMDN1 PE=1 SV=1 |
| Q8N684 | Cleavage and polyadenylation specificity factor subunit 7 OS=Homo sapiens OX=9606 GN=CPSF7 PE=1 SV=1 |
| P30825 | High affinity cationic amino acid transporter 1 OS=Homo sapiens OX=9606 GN=SLC7A1 PE=1 SV=1 |
| Q8N4H5 | Mitochondrial import receptor subunit TOM5 homolog OS=Homo sapiens OX=9606 GN=TOMM5 PE=1 SV=1 |
| Q12769 | Nuclear pore complex protein Nup160 OS=Homo sapiens OX=9606 GN=NUP160 PE=1 SV=3 |
| Q9ULX6 | A-kinase anchor protein 8-like OS=Homo sapiens OX=9606 GN=AKAP8L PE=1 SV=3 |
| P17301 | Integrin alpha-2 OS=Homo sapiens OX=9606 GN=ITGA2 PE=1 SV=1 |
| Q8N697 | Solute carrier family 15 member 4 OS=Homo sapiens OX=9606 GN=SLC15A4 PE=1 SV=1 |
| Q9H3P7 | Golgi resident protein GCP60 OS=Homo sapiens OX=9606 GN=ACBD3 PE=1 SV=4 |
| O94760 | N(G),N(G)-dimethylarginine dimethylaminohydrolase 1 OS=Homo sapiens OX=9606 GN=DDAH1 PE=1 SV=3 |
| Q96G46 | tRNA-dihydrouridine(47) synthase [NAD(P)(+)]-like OS=Homo sapiens OX=9606 GN=DUS3L PE=1 SV=2 |
| Q5BJD5 | Transmembrane protein 41B OS=Homo sapiens OX=9606 GN=TMEM41B PE=1 SV=1 |
| P26572 | Alpha-1,3-mannosyl-glycoprotein 2-beta-N-acetylglucosaminyltransferase OS=Homo sapiens OX=9606 GN=MGAT1 PE=1 SV=2 |
| Q9Y5Q9 | General transcription factor 3C polypeptide 3 OS=Homo sapiens OX=9606 GN=GTF3C3 PE=1 SV=1 |
| Q5RKV6 | Exosome complex component MTR3 OS=Homo sapiens OX=9606 GN=EXOSC6 PE=1 SV=1 |
| O00401 | Neural Wiskott-Aldrich syndrome protein OS=Homo sapiens OX=9606 GN=WASL PE=1 SV=2 |
| Q8NBN7 | Retinol dehydrogenase 13 OS=Homo sapiens OX=9606 GN=RDH13 PE=1 SV=2 |
| O15031 | Plexin-B2 OS=Homo sapiens OX=9606 GN=PLXNB2 PE=1 SV=3 |
| Q9UH99 | SUN domain-containing protein 2 OS=Homo sapiens OX=9606 GN=SUN2 PE=1 SV=3 |
| Q13573 | SNW domain-containing protein 1 OS=Homo sapiens OX=9606 GN=SNW1 PE=1 SV=1 |
| Q8N8A6 | ATP-dependent RNA helicase DDX51 OS=Homo sapiens OX=9606 GN=DDX51 PE=1 SV=3 |
| Q9H307 | Pinin OS=Homo sapiens OX=9606 GN=PNN PE=1 SV=5 |
| A3KN83 | Protein strawberry notch homolog 1 OS=Homo sapiens OX=9606 GN=SBNO1 PE=1 SV=1 |
| Q96CU9 | FAD-dependent oxidoreductase domain-containing protein 1 OS=Homo sapiens OX=9606 GN=FOXRED1 PE=1 SV=2 |
| Q13510 | Acid ceramidase OS=Homo sapiens OX=9606 GN=ASAH1 PE=1 SV=5 |
| Q9UKL0 | REST corepressor 1 OS=Homo sapiens OX=9606 GN=RCOR1 PE=1 SV=2 |
| P11233 | Ras-related protein Ral-A OS=Homo sapiens OX=9606 GN=RALA PE=1 SV=1 |
| Q08170 | Serine/arginine-rich splicing factor 4 OS=Homo sapiens OX=9606 GN=SRSF4 PE=1 SV=2 |
| Q5T8P6 | RNA-binding protein 26 OS=Homo sapiens OX=9606 GN=RBM26 PE=1 SV=3 |
| O15144 | Actin-related protein 2/3 complex subunit 2 OS=Homo sapiens OX=9606 GN=ARPC2 PE=1 SV=1 |
| O15347 | High mobility group protein B3 OS=Homo sapiens OX=9606 GN=HMGB3 PE=1 SV=4 |
| Q8NCA5 | Protein FAM98A OS=Homo sapiens OX=9606 GN=FAM98A PE=1 SV=1 |
| Q8TD30 | Alanine aminotransferase 2 OS=Homo sapiens OX=9606 GN=GPT2 PE=1 SV=1 |
| Q8NF37 | Lysophosphatidylcholine acyltransferase 1 OS=Homo sapiens OX=9606 GN=LPCAT1 PE=1 SV=2 |
| Q96AQ6 | Pre-B-cell leukemia transcription factor-interacting protein 1 OS=Homo sapiens OX=9606 GN=PBXIP1 PE=1 SV=1 |
| Q06265 | Exosome complex component RRP45 OS=Homo sapiens OX=9606 GN=EXOSC9 PE=1 SV=3 |
| Q05519 | Serine/arginine-rich splicing factor 11 OS=Homo sapiens OX=9606 GN=SRSF11 PE=1 SV=1 |
| O15431 | High affinity copper uptake protein 1 OS=Homo sapiens OX=9606 GN=SLC31A1 PE=1 SV=1 |
| O94832 | Unconventional myosin-Id OS=Homo sapiens OX=9606 GN=MYO1D PE=1 SV=2 |
| Q05655 | Protein kinase C delta type OS=Homo sapiens OX=9606 GN=PRKCD PE=1 SV=2 |
| Q96GQ5 | RUS1 family protein C16orf58 OS=Homo sapiens OX=9606 GN=C16orf58 PE=1 SV=2 |
| Q8NCW5 | NAD(P)H-hydrate epimerase OS=Homo sapiens OX=9606 GN=NAXE PE=1 SV=2 |
| Q9H0S4 | Probable ATP-dependent RNA helicase DDX47 OS=Homo sapiens OX=9606 GN=DDX47 PE=1 SV=1 |
| P82663 | 28S ribosomal protein S25, mitochondrial OS=Homo sapiens OX=9606 GN=MRPS25 PE=1 SV=1 |
| Q9H3S7 | Tyrosine-protein phosphatase non-receptor type 23 OS=Homo sapiens OX=9606 GN=PTPN23 PE=1 SV=1 |
| Q96DI7 | U5 small nuclear ribonucleoprotein 40 kDa protein OS=Homo sapiens OX=9606 GN=SNRNP40 PE=1 SV=1 |
| Q9BYD2 | 39S ribosomal protein L9, mitochondrial OS=Homo sapiens OX=9606 GN=MRPL9 PE=1 SV=2 |
| P06865 | Beta-hexosaminidase subunit alpha OS=Homo sapiens OX=9606 GN=HEXA PE=1 SV=2 |
| Q8TED0 | U3 small nucleolar RNA-associated protein 15 homolog OS=Homo sapiens OX=9606 GN=UTP15 PE=1 SV=3 |
| P51151 | Ras-related protein Rab-9A OS=Homo sapiens OX=9606 GN=RAB9A PE=1 SV=1 |
| Q05086 | Ubiquitin-protein ligase E3A OS=Homo sapiens OX=9606 GN=UBE3A PE=1 SV=4 |
| Q99590 | Protein SCAF11 OS=Homo sapiens OX=9606 GN=SCAF11 PE=1 SV=2 |
| Q96SK2 | Transmembrane protein 209 OS=Homo sapiens OX=9606 GN=TMEM209 PE=1 SV=2 |
| P30622 | CAP-Gly domain-containing linker protein 1 OS=Homo sapiens OX=9606 GN=CLIP1 PE=1 SV=2 |
| P35610 | Sterol O-acyltransferase 1 OS=Homo sapiens OX=9606 GN=SOAT1 PE=1 SV=3 |
| P49770 | Translation initiation factor eIF-2B subunit beta OS=Homo sapiens OX=9606 GN=EIF2B2 PE=1 SV=3 |
| Q9Y5Y0 | Feline leukemia virus subgroup C receptor-related protein 1 OS=Homo sapiens OX=9606 GN=FLVCR1 PE=1 SV=1 |
| P26583 | High mobility group protein B2 OS=Homo sapiens OX=9606 GN=HMGB2 PE=1 SV=2 |
| Q9BYD6 | 39S ribosomal protein L1, mitochondrial OS=Homo sapiens OX=9606 GN=MRPL1 PE=1 SV=2 |
| O14745 | Na(+)/H(+) exchange regulatory cofactor NHE-RF1 OS=Homo sapiens OX=9606 GN=SLC9A3R1 PE=1 SV=4 |
| Q92504 | Zinc transporter SLC39A7 OS=Homo sapiens OX=9606 GN=SLC39A7 PE=1 SV=2 |
| O75937 | DnaJ homolog subfamily C member 8 OS=Homo sapiens OX=9606 GN=DNAJC8 PE=1 SV=2 |
| Q8IVD9 | NudC domain-containing protein 3 OS=Homo sapiens OX=9606 GN=NUDCD3 PE=1 SV=3 |
| Q9UIA9 | Exportin-7 OS=Homo sapiens OX=9606 GN=XPO7 PE=1 SV=3 |
| Q9H3P2 | Negative elongation factor A OS=Homo sapiens OX=9606 GN=NELFA PE=1 SV=3 |
| Q96JM3 | Chromosome alignment-maintaining phosphoprotein 1 OS=Homo sapiens OX=9606 GN=CHAMP1 PE=1 SV=2 |
| P05090 | Apolipoprotein D OS=Homo sapiens OX=9606 GN=APOD PE=1 SV=1 |
| Q9BQ52 | Zinc phosphodiesterase ELAC protein 2 OS=Homo sapiens OX=9606 GN=ELAC2 PE=1 SV=2 |
| Q969P0 | Immunoglobulin superfamily member 8 OS=Homo sapiens OX=9606 GN=IGSF8 PE=1 SV=1 |
| O43670 | BUB3-interacting and GLEBS motif-containing protein ZNF207 OS=Homo sapiens OX=9606 GN=ZNF207 PE=1 SV=1 |
| Q9NS86 | LanC-like protein 2 OS=Homo sapiens OX=9606 GN=LANCL2 PE=1 SV=1 |
| Q9H4L5 | Oxysterol-binding protein-related protein 3 OS=Homo sapiens OX=9606 GN=OSBPL3 PE=1 SV=1 |
| P09012 | U1 small nuclear ribonucleoprotein A OS=Homo sapiens OX=9606 GN=SNRPA PE=1 SV=3 |
| Q5HYI8 | Rab-like protein 3 OS=Homo sapiens OX=9606 GN=RABL3 PE=1 SV=1 |
| Q6P4A7 | Sideroflexin-4 OS=Homo sapiens OX=9606 GN=SFXN4 PE=1 SV=1 |
| Q9UHY1 | Nuclear receptor-binding protein OS=Homo sapiens OX=9606 GN=NRBP1 PE=1 SV=1 |
| Q6P6C2 | RNA demethylase ALKBH5 OS=Homo sapiens OX=9606 GN=ALKBH5 PE=1 SV=2 |
| Q9BRP8 | Partner of Y14 and mago OS=Homo sapiens OX=9606 GN=PYM1 PE=1 SV=1 |
| P22607 | Fibroblast growth factor receptor 3 OS=Homo sapiens OX=9606 GN=FGFR3 PE=1 SV=1 |
| Q8NI60 | Atypical kinase COQ8A, mitochondrial OS=Homo sapiens OX=9606 GN=COQ8A PE=1 SV=1 |
| Q13868 | Exosome complex component RRP4 OS=Homo sapiens OX=9606 GN=EXOSC2 PE=1 SV=2 |
| O60318 | Germinal-center associated nuclear protein OS=Homo sapiens OX=9606 GN=MCM3AP PE=1 SV=2 |
| O60256 | Phosphoribosyl pyrophosphate synthase-associated protein 2 OS=Homo sapiens OX=9606 GN=PRPSAP2 PE=1 SV=1 |
| Q5SWX8 | Protein odr-4 homolog OS=Homo sapiens OX=9606 GN=ODR4 PE=1 SV=1 |
| Q14533 | Keratin, type II cuticular Hb1 OS=Homo sapiens OX=9606 GN=KRT81 PE=1 SV=3 |
| Q9Y314 | Nitric oxide synthase-interacting protein OS=Homo sapiens OX=9606 GN=NOSIP PE=1 SV=1 |
| Q5T0W9 | Protein FAM83B OS=Homo sapiens OX=9606 GN=FAM83B PE=1 SV=1 |
| Q9GZV1 | Ankyrin repeat domain-containing protein 2 OS=Homo sapiens OX=9606 GN=ANKRD2 PE=1 SV=3 |
| Q9UFN0 | Protein NipSnap homolog 3A OS=Homo sapiens OX=9606 GN=NIPSNAP3A PE=1 SV=2 |
| Q15067 | Peroxisomal acyl-coenzyme A oxidase 1 OS=Homo sapiens OX=9606 GN=ACOX1 PE=1 SV=3 |
| O14802 | DNA-directed RNA polymerase III subunit RPC1 OS=Homo sapiens OX=9606 GN=POLR3A PE=1 SV=2 |
| Q92466 | DNA damage-binding protein 2 OS=Homo sapiens OX=9606 GN=DDB2 PE=1 SV=1 |
| Q92572 | AP-3 complex subunit sigma-1 OS=Homo sapiens OX=9606 GN=AP3S1 PE=1 SV=1 |
| Q14191 | Werner syndrome ATP-dependent helicase OS=Homo sapiens OX=9606 GN=WRN PE=1 SV=2 |
| O75400 | Pre-mRNA-processing factor 40 homolog A OS=Homo sapiens OX=9606 GN=PRPF40A PE=1 SV=2 |
| Q14241 | Elongin-A OS=Homo sapiens OX=9606 GN=ELOA PE=1 SV=2 |
| Q9BUB7 | Transmembrane protein 70, mitochondrial OS=Homo sapiens OX=9606 GN=TMEM70 PE=1 SV=2 |
| Q9NZC9 | SWI/SNF-related matrix-associated actin-dependent regulator of chromatin subfamily A-like protein 1 OS=Homo sapiens OX=9606 GN=SMARCAL1 PE=1 SV=1 |
| Q9BYG3 | MKI67 FHA domain-interacting nucleolar phosphoprotein OS=Homo sapiens OX=9606 GN=NIFK PE=1 SV=1 |
| P31483 | Nucleolysin TIA-1 isoform p40 OS=Homo sapiens OX=9606 GN=TIA1 PE=1 SV=3 |
| Q8NFZ0 | F-box DNA helicase 1 OS=Homo sapiens OX=9606 GN=FBH1 PE=1 SV=2 |
| P23634 | Plasma membrane calcium-transporting ATPase 4 OS=Homo sapiens OX=9606 GN=ATP2B4 PE=1 SV=2 |
| Q96T76 | MMS19 nucleotide excision repair protein homolog OS=Homo sapiens OX=9606 GN=MMS19 PE=1 SV=2 |
| O95817 | BAG family molecular chaperone regulator 3 OS=Homo sapiens OX=9606 GN=BAG3 PE=1 SV=3 |
| Q5QJE6 | Deoxynucleotidyltransferase terminal-interacting protein 2 OS=Homo sapiens OX=9606 GN=DNTTIP2 PE=1 SV=2 |
| P09429 | High mobility group protein B1 OS=Homo sapiens OX=9606 GN=HMGB1 PE=1 SV=3 |
| P05455 | Lupus La protein OS=Homo sapiens OX=9606 GN=SSB PE=1 SV=2 |
| O75439 | Mitochondrial-processing peptidase subunit beta OS=Homo sapiens OX=9606 GN=PMPCB PE=1 SV=2 |
| P07686 | Beta-hexosaminidase subunit beta OS=Homo sapiens OX=9606 GN=HEXB PE=1 SV=3 |
| P56556 | NADH dehydrogenase [ubiquinone] 1 alpha subcomplex subunit 6 OS=Homo sapiens OX=9606 GN=NDUFA6 PE=1 SV=4 |
| P13010 | X-ray repair cross-complementing protein 5 OS=Homo sapiens OX=9606 GN=XRCC5 PE=1 SV=3 |
| P39687 | Acidic leucine-rich nuclear phosphoprotein 32 family member A OS=Homo sapiens OX=9606 GN=ANP32A PE=1 SV=1 |
| Q9UKM9 | RNA-binding protein Raly OS=Homo sapiens OX=9606 GN=RALY PE=1 SV=1 |
| Q01085 | Nucleolysin TIAR OS=Homo sapiens OX=9606 GN=TIAL1 PE=1 SV=1 |
| Q86U90 | YrdC domain-containing protein, mitochondrial OS=Homo sapiens OX=9606 GN=YRDC PE=1 SV=1 |
| Q15436 | Protein transport protein Sec23A OS=Homo sapiens OX=9606 GN=SEC23A PE=1 SV=2 |
| P12956 | X-ray repair cross-complementing protein 6 OS=Homo sapiens OX=9606 GN=XRCC6 PE=1 SV=2 |
| Q9Y2B0 | Protein canopy homolog 2 OS=Homo sapiens OX=9606 GN=CNPY2 PE=1 SV=1 |
| Q96I24 | Far upstream element-binding protein 3 OS=Homo sapiens OX=9606 GN=FUBP3 PE=1 SV=2 |
| Q15717 | ELAV-like protein 1 OS=Homo sapiens OX=9606 GN=ELAVL1 PE=1 SV=2 |
| Q9UMS4 | Pre-mRNA-processing factor 19 OS=Homo sapiens OX=9606 GN=PRPF19 PE=1 SV=1 |
| Q9UN86 | Ras GTPase-activating protein-binding protein 2 OS=Homo sapiens OX=9606 GN=G3BP2 PE=1 SV=2 |
| O00567 | Nucleolar protein 56 OS=Homo sapiens OX=9606 GN=NOP56 PE=1 SV=4 |
| P29372 | DNA-3-methyladenine glycosylase OS=Homo sapiens OX=9606 GN=MPG PE=1 SV=3 |
| Q13283 | Ras GTPase-activating protein-binding protein 1 OS=Homo sapiens OX=9606 GN=G3BP1 PE=1 SV=1 |
| P55036 | 26S proteasome non-ATPase regulatory subunit 4 OS=Homo sapiens OX=9606 GN=PSMD4 PE=1 SV=1 |
| Q9ULA0 | Aspartyl aminopeptidase OS=Homo sapiens OX=9606 GN=DNPEP PE=1 SV=1 |
| Q96AE4 | Far upstream element-binding protein 1 OS=Homo sapiens OX=9606 GN=FUBP1 PE=1 SV=3 |
| P51991 | Heterogeneous nuclear ribonucleoprotein A3 OS=Homo sapiens OX=9606 GN=HNRNPA3 PE=1 SV=2 |
| Q6PIY7 | Poly(A) RNA polymerase GLD2 OS=Homo sapiens OX=9606 GN=TENT2 PE=1 SV=1 |
| Q9NY33 | Dipeptidyl peptidase 3 OS=Homo sapiens OX=9606 GN=DPP3 PE=1 SV=2 |
| Q9NYF8 | Bcl-2-associated transcription factor 1 OS=Homo sapiens OX=9606 GN=BCLAF1 PE=1 SV=2 |
| Q13148 | TAR DNA-binding protein 43 OS=Homo sapiens OX=9606 GN=TARDBP PE=1 SV=1 |
| Q96EP5 | DAZ-associated protein 1 OS=Homo sapiens OX=9606 GN=DAZAP1 PE=1 SV=1 |
| Q13435 | Splicing factor 3B subunit 2 OS=Homo sapiens OX=9606 GN=SF3B2 PE=1 SV=2 |
| Q14254 | Flotillin-2 OS=Homo sapiens OX=9606 GN=FLOT2 PE=1 SV=2 |
| P22626 | Heterogeneous nuclear ribonucleoproteins A2/B1 OS=Homo sapiens OX=9606 GN=HNRNPA2B1 PE=1 SV=2 |
| P07910 | Heterogeneous nuclear ribonucleoproteins C1/C2 OS=Homo sapiens OX=9606 GN=HNRNPC PE=1 SV=4 |
| Q9HBH5 | Retinol dehydrogenase 14 OS=Homo sapiens OX=9606 GN=RDH14 PE=1 SV=1 |
| Q1KMD3 | Heterogeneous nuclear ribonucleoprotein U-like protein 2 OS=Homo sapiens OX=9606 GN=HNRNPUL2 PE=1 SV=1 |
| Q96DH6 | RNA-binding protein Musashi homolog 2 OS=Homo sapiens OX=9606 GN=MSI2 PE=1 SV=1 |
| Q9NVI1 | Fanconi anemia group I protein OS=Homo sapiens OX=9606 GN=FANCI PE=1 SV=4 |
| Q8ND24 | RING finger protein 214 OS=Homo sapiens OX=9606 GN=RNF214 PE=1 SV=2 |
| Q9Y6K5 | 2'-5'-oligoadenylate synthase 3 OS=Homo sapiens OX=9606 GN=OAS3 PE=1 SV=3 |
| P36957 | Dihydrolipoyllysine-residue succinyltransferase component of 2-oxoglutarate dehydrogenase complex, mitochondrial OS=Homo sapiens OX=9606 GN=DLST PE=1 SV=4 |
| Q14764 | Major vault protein OS=Homo sapiens OX=9606 GN=MVP PE=1 SV=4 |
| Q9BV40 | Vesicle-associated membrane protein 8 OS=Homo sapiens OX=9606 GN=VAMP8 PE=1 SV=1 |
| P26368 | Splicing factor U2AF 65 kDa subunit OS=Homo sapiens OX=9606 GN=U2AF2 PE=1 SV=4 |
| Q9UIJ7 | GTP:AMP phosphotransferase AK3, mitochondrial OS=Homo sapiens OX=9606 GN=AK3 PE=1 SV=4 |
| Q9Y4C8 | Probable RNA-binding protein 19 OS=Homo sapiens OX=9606 GN=RBM19 PE=1 SV=3 |
| Q13151 | Heterogeneous nuclear ribonucleoprotein A0 OS=Homo sapiens OX=9606 GN=HNRNPA0 PE=1 SV=1 |
| Q01844 | RNA-binding protein EWS OS=Homo sapiens OX=9606 GN=EWSR1 PE=1 SV=1 |
| Q14444 | Caprin-1 OS=Homo sapiens OX=9606 GN=CAPRIN1 PE=1 SV=2 |
| Q07866 | Kinesin light chain 1 OS=Homo sapiens OX=9606 GN=KLC1 PE=1 SV=2 |
| Q8TEM1 | Nuclear pore membrane glycoprotein 210 OS=Homo sapiens OX=9606 GN=NUP210 PE=1 SV=3 |
| Q9Y2L1 | Exosome complex exonuclease RRP44 OS=Homo sapiens OX=9606 GN=DIS3 PE=1 SV=2 |
| Q99798 | Aconitate hydratase, mitochondrial OS=Homo sapiens OX=9606 GN=ACO2 PE=1 SV=2 |
| O75955 | Flotillin-1 OS=Homo sapiens OX=9606 GN=FLOT1 PE=1 SV=3 |
| Q13136 | Liprin-alpha-1 OS=Homo sapiens OX=9606 GN=PPFIA1 PE=1 SV=1 |
| Q86V81 | THO complex subunit 4 OS=Homo sapiens OX=9606 GN=ALYREF PE=1 SV=3 |
| Q9UGI8 | Testin OS=Homo sapiens OX=9606 GN=TES PE=1 SV=1 |
| P55265 | Double-stranded RNA-specific adenosine deaminase OS=Homo sapiens OX=9606 GN=ADAR PE=1 SV=4 |
| Q15427 | Splicing factor 3B subunit 4 OS=Homo sapiens OX=9606 GN=SF3B4 PE=1 SV=1 |
| Q9P015 | 39S ribosomal protein L15, mitochondrial OS=Homo sapiens OX=9606 GN=MRPL15 PE=1 SV=1 |

**TableS6. The primer sequences.**

| circMALAT1-forward primer | AGCTGAGTGATAAAGGCTGAGTG |
| --- | --- |
| circMALAT1-reverse primer | TGATCTGGTCCATTAAAGAGTGTTC |
| linear MALAT1-forward primer | TGTCTGCGAACACTCTTT |
| linear MALAT1-reverse primer | AATCTCCCACCTGTCTAA |
| β-actin-forward primer | GAAGGTGACAGCAGTCGGTT |
| β-actin-reverse primer | GGACTTCCTGTAACAACGCA |
| lncRNA-NEAT1-forward primer | TTTGTGCTTGGAACCTTGCT |
| lncRNA-NEAT1-reverse primer | TCAACGCCCCAAGTTATTTC |
